# MICROPHERRET: MICRObial PHEnotypic tRait ClassifieR using Machine lEarning Techniques

**DOI:** 10.1186/s40793-024-00600-6

**Published:** 2024-08-08

**Authors:** Edoardo Bizzotto, Sofia Fraulini, Guido Zampieri, Esteban Orellana, Laura Treu, Stefano Campanaro

**Affiliations:** Department of Biology, University of Padova, Padova, 35131 Italy

**Keywords:** Functional classification, Machine learning, Microbial genome, Metagenome, Methanogenesis

## Abstract

**Background:**

In recent years, there has been a rapid increase in the number of microbial genomes reconstructed through shotgun sequencing, and obtained by newly developed approaches including metagenomic binning and single-cell sequencing. However, our ability to functionally characterize these genomes by experimental assays is orders of magnitude less efficient. Consequently, there is a pressing need for the development of swift and automated strategies for the functional classification of microbial genomes.

**Results:**

The present work leverages a suite of supervised machine learning algorithms to establish a range of 86 metabolic and other ecological functions, such as methanotrophy and plastic degradation, starting from widely obtainable microbial genome annotations. Tests performed on independent datasets demonstrated robust performance across complete, fragmented, and incomplete genomes above a 70% completeness level for most of the considered functions. Application of the algorithms to the Biogas Microbiome database yielded predictions broadly consistent with current biological knowledge and correctly detecting functionally-related nuances of archaeal genomes. Finally, a case study focused on acetoclastic methanogenesis demonstrated how the developed machine learning models can be refined or expanded with models describing novel functions of interest.

**Conclusions:**

The resulting tool, MICROPHERRET, incorporates a total of 86 models, one for each tested functional class, and can be applied to high-quality microbial genomes as well as to low-quality genomes derived from metagenomics and single-cell sequencing. MICROPHERRET can thus aid in understanding the functional role of newly generated genomes within their micro-ecological context.

**Supplementary Information:**

The online version contains supplementary material available at 10.1186/s40793-024-00600-6.

## Background

Genome-centric metagenomics allows the reconstruction of draft genomes of single microorganisms called metagenome-assembled genomes (MAGs) [1]. Analysis of diverse MAGs from both phylogenetic and functional perspectives enables a comprehensive exploration of the taxonomic composition and functional processes within the sampled microbial community [2, 3]. From an ecological standpoint, metagenomics studies offer invaluable insights into ecosystem functions and responses to environmental dynamics [4]. Associations of microorganisms to specific functional roles are pivotal for understanding their role in the community, and to elucidate ecological niches [5–7]. Consequently, predicting the functional association of organisms from metagenomic data emerges as a critical task in community analysis. Advancements in sequencing technologies and metagenomics, together with the increase in the number of metagenomic studies, led to a massive generation of sequencing data from various microbial communities. Large-scale initiatives such as the Tara Oceans Project [8], the Earth Microbiome Project [9], and the Unified Human Gastrointestinal Genome [10] have significantly expanded our understanding through in-depth sequencing of microbial communities. Additionally, there is an increasing capacity for obtaining a high number of Single-Amplified Genomes (SAGs) from single microbial cells through single-cell sequencing techniques [11]. The abundance of data obtained resulted in the creation of databases of MAGs and SAGs from different environments such as the genomes from the Earth Microbiome Project, Ocean Microbiomics Database, and the Human Gastrointestinal Genome catalog [12–14].

The high number of MAGs obtained from metagenomic experiments, and the issues associated with their quality, represent a challenge for manual functional association, necessitating the development of specialized bioinformatics tools [15–17]. The functional annotation of MAGs typically involves the identification of specific genes that are recognized for their association with distinct functions or metabolic pathways [10–12]. This process often relies on information sourced from databases such as the Kyoto Encyclopedia of Genes and Genomes (KEGG) [18]. Since the genomic sequences are frequently incomplete, it could be hard to define functional associations based on the identification of complete KEGG functional pathways. The efficacy of genomic annotations and subsequent mapping onto databases is contingent upon the availability of functional information, thereby limiting its applicability to well-known microbial communities. Moreover, information on annotated genes may be absent from databases, and genes associated with unknown functions might lack categorization [19]. Additionally, genes crucial for a particular function may belong to pathways not directly linked to that function or they can lack functional annotation [20]. Thus, tools such as METABOLIC [16] and NCycDB [21] are widely used and map the proteins predicted from MAGs onto several databases to recover the annotations and to infer functional traits related to metabolites. These tools are very efficient when the traits are associated with well-defined metabolic pathways, but they fall short when the target is to discover functions that are not associated with existing KO codes or cannot infer them if genes are missing. Another possible strategy is to exploit the knowledge gathered in the last few years into extensive databases for functional association [17]. These databases store functional information of several phenotypic traits linked to many different microbes, allowing the connection of the stored organisms to their metabolic or functional traits [17, 22, 23]. These collections of data mostly result from literature-based assignments and can either be specific for a metabolic process of interest or general, containing information on several different metabolic/functional traits [24, 25]. One prominent example is the Functional Annotation of Prokaryotic Taxa (FAPROTAX), a literature-based database designed for the functional classification of taxa [26]. It currently includes 92 phenotypic traits associated with more than 5,000 distinct taxonomic entries, and it has been used in several studies as a source of microbial functional information, and to classify organisms into functional groups [27–30]. The functions within FAPROTAX predominantly encompass metabolic functions attributed to microbial species, such as their ability to use, convert, or produce specific compounds. Notably, while FAPROTAX offers a valuable resource for associating microbial species with their functional capabilities, the analysis is challenging. The process can be prone to inaccuracies, time-consuming, and computationally demanding due to the high dimensionality of the dataset. For example, the analysis of species not present in the database, such as the majority of MAGs from metagenomics studies, is unfeasible and there may be cases where genomes that are present may not contain genes for particular functions due to deletions or can have additional genes for functions not explored in FAPROTAX. Moreover, the database by itself is a text file that is hard to query and contains outdated taxonomic names for certain species. Thus, verifying whether a genome possesses a particular function is not always straightforward and may require extensive analyses. In the case of MAGs, which frequently have incomplete genomes, entire pathways could not be present due to the intrinsic limitations of the approach.

Consequently, alternative approaches must be explored to efficiently harness and glean insights from the vast knowledge stored in FAPROTAX in a faster and more user-friendly manner.

The application of supervised machine learning in diverse biological domains, including genomics, evolutionary biology, ecology, structural biochemistry, and drug discovery, has been instrumental for predictive purposes [31–33]. Within this framework, machine learning methods have proven effective in functionally classifying organisms based on their genomic content [34–36]. Notably, these techniques exhibit enhanced predictive efficiency for metabolic traits in non-model organisms compared to constraint-based modeling [37, 38]. Indeed, this approach does not need prior knowledge about the non-model organisms, since the classifier learns the required information for the prediction from data of the model organisms used during the training step. To obtain efficient models, extended datasets for training are needed. The high amount of data stored in FAPROTAX is suitable to be used as a training set for machine learning algorithms to obtain a tool that classifies organisms according to their FAPROTAX functional group.

This approach was previously undertaken by Farrell and colleagues [34], who integrated FAPROTAX functional associations with a set of NCBI genomes in a dataset comprising 9407 samples. Through these data, they devised a machine learning approach to infer phenotypic traits from genomic annotations, successfully identifying genetic markers associated with phenotypic functions stored in FAPROTAX. The tool demonstrated efficacy by accurately classifying 65 phenotypic traits across large-scale metagenomic datasets from diverse environments. However, the strategy applied to choose the best supervised machine learning algorithm might not have been optimal. The choice was based on the model that generally outperformed others across all trained classifiers, potentially overlooking subtle performance variations within each functional group. Furthermore, the absence of an optimization step for model hyperparameters may have influenced classifier performance. Importantly, the FAPROTAX dataset was solely employed as a source of functional information for genomes collected from NCBI, neglecting its potential as a reservoir of genetic information. Since the taxa-function links in the database imply that every organism in a taxon can perform the associated functions, a more effective approach would involve initially compiling the taxonomic groups from FAPROTAX and collecting genomes from these taxa across various public databases. By training the models on the retrieved genomes it would be possible to increase the available data and fully leverage the information stored in the FAPROTAX database.

Building on the promising finding described above, the primary objective of this work is to develop a tool dedicated to inferring microbial functional traits from vectors of gene annotations. The approach involves the optimization of various supervised machine learning algorithms, fine-tuned and trained using data extracted from the FAPROTAX database. The end goal is to systematically classify organisms into functions and evaluate the efficacy of the selected models in handling incomplete genomes. The tool was employed to predict the functions of MAGs previously recovered from anaerobic digestion environments, showcasing its applicability in unraveling the functional attributes of microbial communities within specific ecological niches and providing practical insights into the functional dynamics of microbial communities.

## Methods

### Dataset generation

A comprehensive representation of the dataset generation procedure is provided in Figure S1. To extract relevant information from the FAPROTAX database (version 1.2.6), a custom FAPROTAX parser written in Python was developed. The FAPROTAX information, stored in a plain text file (txt), featured 92 functions, i.e. the name of the functional group, followed by a list of affiliated taxa. Each group entry in the database consists of a list of descending taxonomic levels separated and delimited by asterisks (“*”) characters. The asterisks indicate that there might be higher or lower taxonomic levels that have been included in the name. The database allows overlaps between groups and the presence of duplicate entries within a group. Set operations like sum, subtraction, and intersection are supported between functional groups, generating a hierarchical structure of the database. The script, taking the .txt file as input, identified functional group names and stored associated entries without repetitions in a dictionary. This process yielded a 5,008 × 92 matrix, with taxonomic groups as rows, functional groups as columns, and binary values (“1” or “0”) indicating membership in functional groups. This list of unique taxonomic entries was used to associate FAPROTAX taxonomic units with their NCBI ID [39]. This step was crucial to retrieve the genomes of organisms stored in FAPROTAX from the NCBI microbial genome database, but it was hindered by several factors, including typos, the use of legacy taxonomy, and the frequent updates of the NCBI taxonomy database which sometimes make the stored taxonomy obsolete. Information such as organisms’ IDs, ranks and lineages was retrieved from files downloaded from the NCBI taxonomy database. Different strategies were implemented to couple the taxonomy names to NCBI IDs, searching for perfect and imperfect matches between the database entries and the taxonomic groups stored in NCBI files. Among the 5,008 database entries, 513 referred to the same organisms, and redundancy was removed by considering the entries only once. The resulting IDs were combined with the ones obtained from searching the list of 5,008 unique database entries on the NCBI taxonomy browser. Some of the undetected IDs were manually searched and checked. 4,732 taxa were successfully identified, while the remaining 276 could not be detected because of typos or because they referred to obsolete NCBI versions. Identifiers wrongly associated with more than one entry were also present. In total, 4,214 unique IDs were retrieved.

With the obtained NCBI IDs, genomes associated with taxa were downloaded from the NCBI database to construct a custom dataset for training the machine learning tool. The FAPROTAX database includes entries at several different taxonomic ranks: among the detected 4,214 unique NCBI IDs, 3,268 were associated with species or lower ranks (subspecies, serotype, strain, and isolate), but IDs referring to higher taxonomic levels were also frequently present (Table S1).

The script gimme_taxa.py, from ncbi-genome-download (version 0.3.3) [40], was used to fetch all the descendants’ taxa IDs of the 3,382 IDs found as perfect matches between FAPROTAX and NCBI entries.

The remaining IDs were kept aside for validation purposes. The script returned 115,446 descendant IDs, with a subsequent check revealing 34,441 genomes in the RefSeq database, including 4,329 complete genomes. A Python pipeline was developed to investigate the taxonomic distribution of the available genomes at any given taxonomy level. The script associated each genome with its NCBI ID and with its rank and traced back the ancestors with the desired rank. The script returned outputs describing the distribution of the genomes at the given rank, as a file with the percentage of genomes sharing a common ancestor. Investigating the distribution of the number of genomes per species revealed that most of the species (98.9%) were characterized by a maximum of 30 genomes, with only 84 species with more genomes. Thus, a threshold of 30 genomes per species was set for downloading. These genomes were chosen at random from the available ones, prioritizing complete genomes if available, selecting 14,725 for the download. Quality control was performed by CheckM2 [41], to assess microbial genome quality, and genomes were filtered removing those with completeness lower than 90% and contamination higher than 5% [42]. The remaining 14,364 genomes were annotated by eggNOG-mapper (version 2.1.10) [43] to obtain the list of KEGG orthologs mapped to each genome. The gene finding step was performed by using Prodigal [44]. A dataset composed of two matrices was created: a features (KOs) matrix, which stored the genomic information as the copy number of the KOs, and a label (function) matrix, which associated each genome to the FAPROTAX functional groups.

### Machine learning classifiers

This study aimed to train a supervised machine learning tool with as many classes as the number of FAPROTAX groups that allowed each genome to perform multiple functions. Thus, separate binary classifiers were trained for all the classes. Here, we use the term “functional class” to refer to the set of genomes associated with each specific function. Functional classes were used in the construction of function-specific binary classifiers: genomes belonging to a class served as positive labels for training the associated model, while the remaining genomes in the custom dataset served as the negative ones. Functional classes differ from FAPROTAX functional groups, which consist of taxonomic units rather than genomes. Each functional class was derived from extracting information from the corresponding FAPROTAX group as described above.

Three FAPROTAX groups, namely “nonphotosynthetic cyanobacteria,” “anammox,” and “chloroplasts,” were excluded due to the limited sample size of their corresponding classes (less than 3 genomes). Eighty-nine function-specific classifiers were trained on 80% of the dataset created from the FAPROTAX database (training set) and their performances were evaluated on the remaining 20% (test set) (Figure S2) [45].

Normalization of datasets, involving KO copy numbers, was performed to enhance model performance by converting them into normally distributed data with zero mean and unit variance during the preprocessing stage.

Given the dataset’s high imbalance, genomes were distributed between the train and test sets in a stratified manner. Evaluation metrics, particularly Matthew’s correlation coefficient (MCC), were employed for their reliability in assessing both balanced and imbalanced datasets [46, 47]. Three supervised machine learning algorithms—logistic regression (LR), random forest (RF), and support vector machines (SVM)—were implemented using the Python scikit-learn library (version 1.2.2) [48], along with a neural network algorithm implemented using Keras (version 2.11.0) [49]. The training process involved the development of 89 function-specific classifiers, each evaluated on the corresponding test set. The best-performing model for each class (Table S2), based on the highest MCC score, was selected and stored in .sav files for sklearn implementations and .hdf5 for the neural network.

A nested cross-validation procedure was performed for LR, RF, and SVM on the train set to reduce the bias in combined hyperparameter (HP) tuning and model evaluation. This approach consists of two nested CV loops, HP selection is performed in the inner one, while the outer one computes an unbiased estimate of the expected accuracy of the algorithm. The procedure returns the performance score of *n* trained and tuned models. Results can be compared to select the optimized model.

HP were tuned with the grid search approach, by evaluating all the possible combinations of a given set of HP and selecting the combination with the highest evaluation score.

In LR models, the regularization strength (C) and penalty were optimized (Table S2). For RF, the grid search procedure was applied for tuning the employed number of used trees and the size of the random subsets of features to consider when splitting a node of the decision trees. Finally, the kernel and penalty value (C) were tuned for SVM. 3-fold stratified cross-validation was used both on the outer and inner loops. A three-element list of best HP combinations was returned, along with their average MCC scores calculated during the grid search procedure and their MCC scores obtained from testing the model on the *k* set in the outer loop. These results were compared to choose the best HP combination.

For neural networks, the hyperparameter optimization process was carried out using 80% of the total FAPROTAX dataset by testing different combinations of Dropout values and the number of Units in the Dense layer. The optimization was carried out 5 times and the best set of hyperparameter combinations was selected based on the best results on the 20% of the remaining dataset, as previously described for the other models. The chosen hyperparameters were used on the whole training set to get the final MCC value of the model.

Function-specific models were grouped at a higher level to investigate the performance of the tool in a more biologically contextualized manner. The 89 predicted functions were clustered into 7 superclasses with different sizes: “carbon metabolism”, “nitrogen metabolism”, “sulfur metabolism”, “parasites or symbionts”, “phototrophy”, “arsenic ions metabolism” and “metals metabolisms”. 4 functions were not grouped due to their peculiar characteristics. The evaluation metrics for each functional superclass were obtained by combining predictions and observations from the individual models to calculate a comprehensive value.

In this project, feature importance (FI) was computed to infer the relationship between gene KOs and functions and to retrieve the more relevant KOs for the classification [50]. Model-dependent methods were used to extract the importance scores directly from the models. In logistic regression, the weights or coefficients of the model features were retrieved and the coefficients with scores different than 0, indicating that they affected the classification process, were stored. The number of resulting KOs varied greatly according to the penalty used, which was chosen in the HP tuning step. In the RF classifiers, scores representing the features’ relative importance were directly extracted from the models. These were estimated by the expected fraction of samples in which a feature contributes to the final prediction decision. In SVM models, feature importance extraction strongly depends on the used kernel. In linear kernel algorithms, it was possible to retrieve the trained linear weights associated with features as a measure of their importance. On the other hand, models with non-linear kernels did not allow extracting this information since the data are transformed by the kernels into a space different from the input one. To retrieve the desired scores without employing time-consuming approaches, SVM models with linear kernels were exclusively trained to obtain the list of the features trained linear coefficients. NN models were analyzed with SHAP [51] however, it was not possible to retrieve meaningful genes from them due to the large number of features. Considering that SHAP returns the importance of each feature for each prediction, there was not a consensus on the main KOs contributing to the classification. To obtain the list for all the classifiers for comparison, the second best trained algorithm was used for the functions trained with NN. The resulting lists of ranked KOs per classifiers were filtered for scores different from 0 (Table S3). If the number of features was too high to be analyzed, only 10% of KOs at the top and bottom of the lists were investigated.

### Tool validation

The final tool underwent validation across three distinct datasets to ensure its robustness and reliability. First, MICROPHERRET was tested on an independent set of complete genomes from the FAPROTAX database; these genomes were retrieved from the 1,350 NCBI IDs which were associated with the database taxa as non-perfect matches and were not included in the initial dataset creation process (Figure S1). The genome filtering and download processes previously reported were applied, and overlaps between the detected genomes and the training dataset were checked. 4,146 complete genomes were obtained and associated with the FAPROTAX functional groups for comparison with the classifiers’ results (Table S2).

Moreover, a dataset of simulated fragmented genomes (SGF) was created by randomly fragmenting the collected 4,146 genomes to test MICROPHERRET performance on metagenomic data. A previously developed Python script (De Bernardini N., personal communication) was used for the fragmentation procedure. The tool fragments genomes by removing random sequences of selected lengths sampled from a normal distribution until the desired completion percentage is achieved. Genome completion is estimated with respect to the species’ genome size, thus not considering the direct effect on gene content. Three separate datasets were obtained, based on the target percentage of completeness: 90%, 70%, and 50%, corresponding to the 10%, 30% and 50% of genome length removed.

MICROPHERRET was further tested on the Biogas Microbiome database, composed of 4,568 metagenomes assembled genomes from 314 samples from anaerobic digestion environments [52, 53]. 635 MAGs were found to be present in FAPROTAX and were associated with their functional groups by using taxonomic information only. These MAGs were used for validation purposes (Table S4), while the remaining ones were excluded since the missing association with the database groups did not allow information to be used as a comparison with the prediction results. However, the tool was also used on the remaining portion of the dataset to predict the associated functions.

Each dataset was functionally annotated with eggNOG-mapper and was made consistent with the training set of the final tool by adjusting the number of KOs to match. Subsequently, the datasets were scaled and trained with function-specific scalers and classifiers. If functions associated with genomes were known, the MCC and confusion matrix of each classifier were calculated to evaluate the performance on the validation sets.

The computational time required for training the entire 89-function specific classifiers on the largest used validation set (4,146 complete genomes) was 1 h 9 min and 41 s (4,181 s), with an average of ≈ 1 predicted genome per second. The measured peak memory was 6.1 Gbyte. The analysis was performed using a 12th Gen Intel(R) Core(TM) i9-12900KF CPU with 32GB of RAM.

### Pipeline to refine functional classifiers

A pipeline to allow the creation of a curated classifier for a class of interest was developed. The classifier is trained on a modified version of the FAPROTAX database, with genomes associated with the target function provided directly as input to the pipeline. The script takes as input the name of the functional class for training the classifier, and the folder containing the genomes belonging to the class annotated by eggNOG-mapper. If the given functional class is already present in the training set, the previously associated genomes are removed, and replaced by those provided by the user. Moreover, if the genomes provided by the user as part of the new class are already present in the training set from FAPROTAX, they are removed from the dataset and left only in the new class. This precaution is taken to prevent these genomes from being part of both the set associated with the function and not associated with the function. Such a situation could introduce a misleading signal for the model and potentially impede accurate classification.

Genomes associated with the function might not be used to create the new classifier class, e.g., some genomes may be kept aside for validation. The eggNOG-mapper output files are used to build the feature matrix with KOs as columns and genomes as rows. The newly created training set is then used to train the new classifier with the same methods explained above. The new classifier is provided as output by the pipeline, ready to be used for predicting whether the input species of interest can perform the newly defined function.

### Case study: acetoclastic methanogenesis

The described pipeline was used to create a curated classifier for the functional class “acetoclastic methanogenesis”. Fifteen genomes belonging to the *Methanosarcina*, *Methanothrix*, and *Methanocalculus* genera were included in the class. The genomes were annotated with eggNOG-mapper and the output files were provided as input to the pipeline. A list of genomes of 17 *Methanosarcina* species associated with the function, which were kept aside for validation purposes, was removed from the original training set. Among these 17 genomes, 9 were positively associated with the FAPROTAX group “methanogenesis by disproportionation of methyl groups” but were erroneously not included in the acetoclastic class. This was a consequence of the structures of these two groups in FAPROTAX, where the “acetoclastic methanogenesis” group consisted of four *Methanosarcina* species and the entire *Methanosaetaceae* family, while the “methanogenesis by disproportionation of methyl groups’’ group was characterized by the entire *Methanosarcinaceae*. The new dataset included a total of 14,352 genomes and 11,470 KOs, with the provided 15 genomes positively associated with the function, and the remaining ones from the FAPROTAX-created dataset as negatively associated. The curated classifier was utilized on the two previously employed datasets, namely the collection of 4,146 genomes obtained from FAPROTAX and the Biogas Microbiome database. Additionally, it was applied to a custom-created dataset, comprising the mentioned 17 genomes of *Methanosarcina* associated with acetoclastic methanogenesis, and 100 genomes of organisms engaged in cellulolysis, fermentation, sulfate reduction, and hydrogenotrophic methanogenesis. The genomes of organisms performing these functions were sourced from the BacDive database and annotated using eggNOG-mapper.

## Results

### Genomic and functional data characterization

**Fig. 1:**
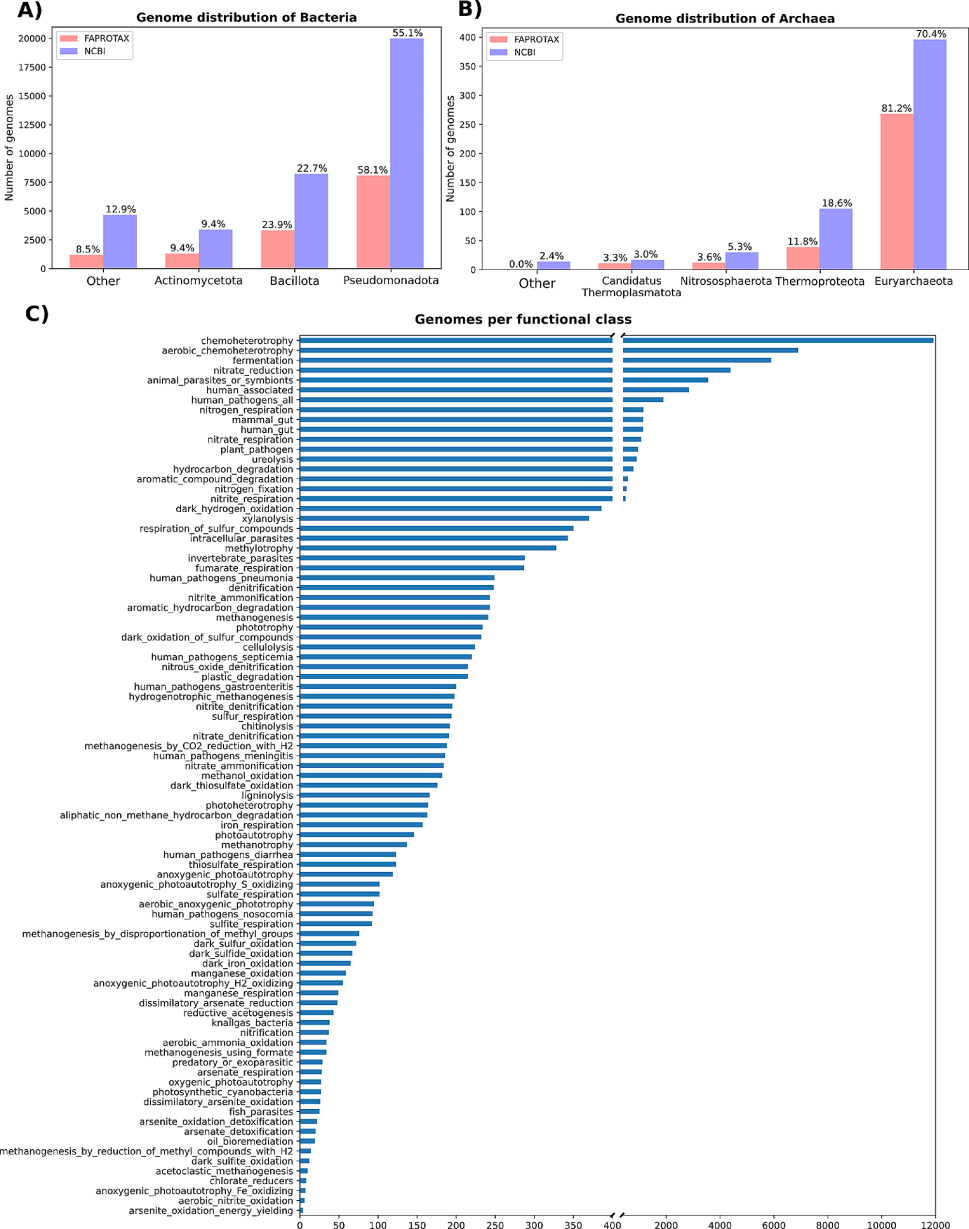
Number of genomes associated with different bacteria (**A**) and archaeal (**B**) phyla in FAPROTAX and NCBI databases. Values on top of the bars represent the percentage of genomes over the total number of the database. (**C**) Number of genomes associated with specific functions. A single genome can be linked with more than one function

In order to develop classifiers for inferring functions, a custom dataset with genomes and information on their functions was developed for training the machine learning tool. The FAPROTAX database was used in a two-step process as a source of information. Initially, taxonomic groups within the database were employed to assemble the dataset by fetching corresponding genomes from the NCBI database [54] (cited December 2023). Subsequently, the detected taxa and the FAPROTAX functional groups were associated with their corresponding entry in the database (Fig. 1).

By parsing the FAPROTAX database it was found that it encompassed 5,008 taxonomic groups, unevenly distributed across 92 functional groups. Most of the database entries (94.5%) were successfully assigned to NCBI IDs in order to fetch and download the genomes from NCBI (Figure S3). Starting from 3,382 NCBI IDs at different taxonomic levels, a total of 14,725 genomes were recovered (Figure S1), representing 7,948 species spanning 39 phyla.

Quality assessments of the downloaded genomes, taking into account contamination and completeness levels, revealed that 14,364 genomes met the defined quality criteria, with completeness higher than 90% and contamination lower than 5% (Figure S4). The remaining genomes were excluded from the analysis. The 14,364 high quality genomes underwent gene finding and the predicted proteins were functionally annotated with eggNOG-mapper [43], resulting in the retrieval of lists of KEGG Orthologs (KOs) associated with each genome (Figure S1).

Analysis of the taxonomic distribution within the custom dataset revealed the presence of 14,037 bacterial and 327 archaeal genomes. The acquired data facilitated the creation of the dataset for training and evaluating the machine learning model, comprising KOs as features and the functions associated as label matrices. Analysis of the feature matrix showed that 11,469 KOs were annotated to the genomes. The number of genes annotated by at least a KO ranged from 24 to 99%, with a mean KO coverage of 40%.

The structure of FAPROTAX groups, together with problems in entry-ID associations and availability of some genomes in the NCBI database caused two classes, “chloroplast” and “nonphotosynthetic cyanobacteria”, to be empty, while the “anammox” class was characterized by a single genome. Overall, the FAPROTAX-derived dataset offers a diverse and representative sample for functional trait inference in microbial communities.

### Machine learning model evaluation

To infer microbial functional traits from the genome content, MICROPHERRET was developed by combining different supervised machine learning algorithms. The tool consisted of 89 function-specific binary classifiers, tuned and trained on the custom dataset represented by the 14,364 genomes obtained from the FAPROTAX database. Each classifier can predict the association of an organism to a function of interest using as input the KEGG annotation of all its genes and also the copy number of each KO ID. Classifier performance was evaluated using Matthew’s Correlation Coefficient (MCC), and a classifier was considered successful when its MCC exceeded 0.7. The global mean MCC for each algorithm was obtained, indicating the support vector machines (SVM) as the best method (0.83), followed by logistic regression (LR) with 0.81 MCC, and random forest (RF) with 0.79 MCC. Neural networks (NN) performed the worst out of all the classifiers with 0.69 MCC.

**Fig. 2:**
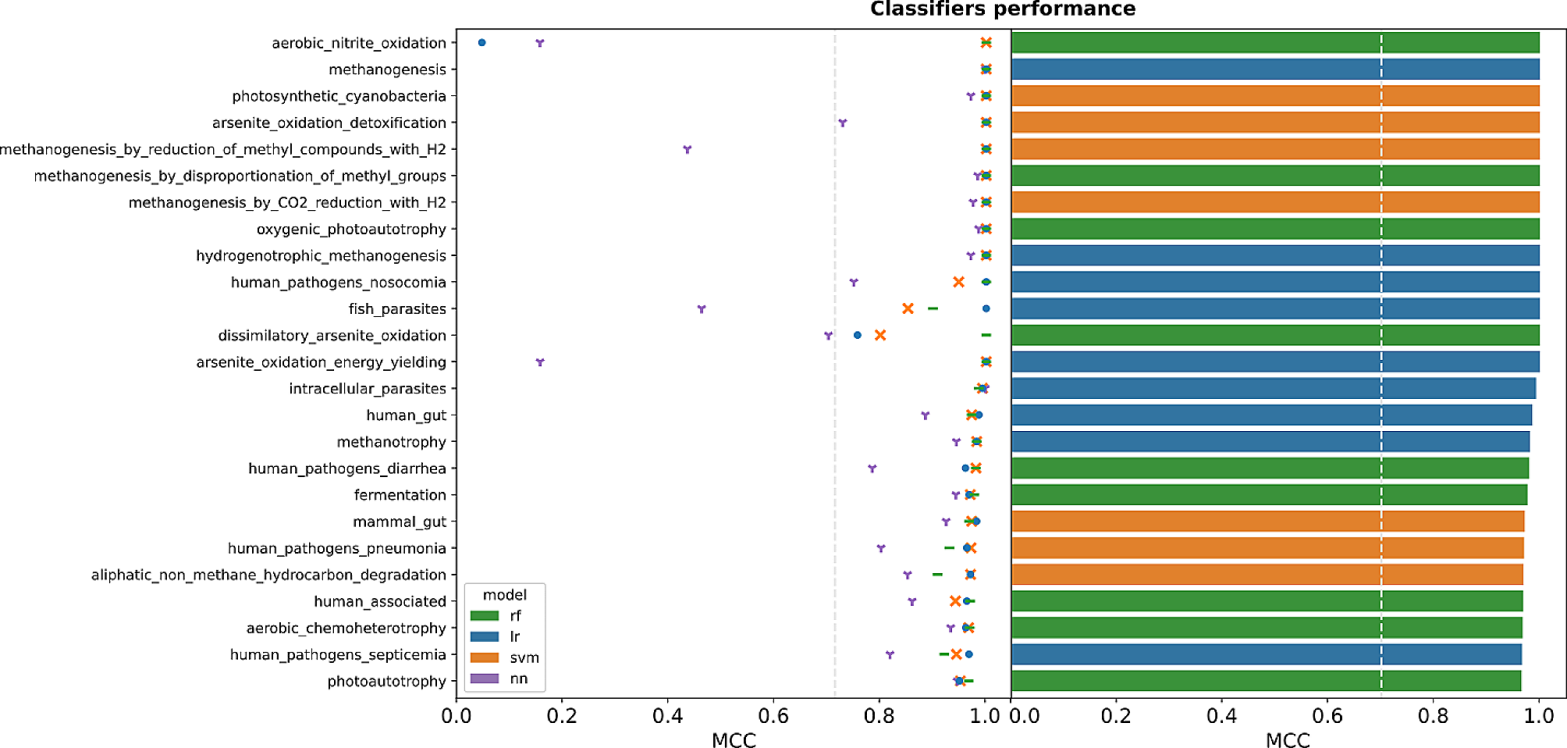
MCC scores on the test sets of the four applied algorithms and MCC score of the selected best algorithm per functional class. Only the 25 classes with top-performing models are included in the plot, while the full results are reported in Figure S5

For each functional class, the performance scores on the test sets of the tuned algorithms were compared to identify the most efficient approach to be included in the tool. In most cases, the three conventional machine learning methods obtained similar MCC values, but there were exceptions (Fig. 2, Figure S5). For example, the “aerobic nitrite oxidation” model obtained high classification performance for RF and SVMs, while LR performance was as good as a random classifier. The performance of NN models was similar in most cases, although with MCC values slightly lower than the other techniques employed. On the other hand, for three classes, “dark sulfite oxidation”, “oil bioremediation”, and “anoxygenic photoautotropy Fe oxidizing”, the NN classifier obtained a MCC value higher than the traditional counterpart which was not able to be trained properly on these functions (Figure S5).

The majority of models were successful, with 81 out of 89 classifiers achieving an MCC greater than 0.7. Among the remaining 8 classifiers, 5 attained MCC values higher than 0.5. Notably, three classifiers, “anoxygenic photoautotrophy Fe oxidizing”, “dark sulfite oxidation” and “oil bioremediation,” exhibited low MCC values, with 0.39, 0.24, and 0.09, respectively. The models’ performance showed no correlation with the average KO coverage of the genomes in the corresponding functional class (Spearman’s correlation coefficient ≈ 0.14, *p*-value ≈ 0.18, Figure S6).

Among all the best classifiers for each functional class, 23 were LR models, 30 were RF models, 29 were SVM models and 7 were NN models (Table S2). However, it is worth noting that for many classifiers, the overall difference between models was very small (Figure S5). The scores on the test sets were consistent with the validation performance scores for most classifiers. The predicted functions were grouped at a higher functional level according to their biological role to provide a general indication of which general phenotypes are predicted better by the tool (Table S5). Among the resulting 7 functional superclasses, “carbon metabolism” is associated with the highest MCC (≈ 0.97), followed by “parasites or symbionts” (≈ 0.94). In general, the tool obtained high performances in 5 superclasses (MCC > 0.7), while it was less efficient in the prediction of sulfur-related (≈ 0.57) and phototrophy-related functions (≈ 0.37).

On the test sets, there is no correlation between the number of genomes associated with classifiers and the performance efficiency (measured as MCC) (Spearman’s correlation coefficient ≈ 0.15, *p*-value = 0.17, Figure S7A).

The list of ortholog genes employed by the models for classification was extracted to infer genes linked to the analyzed phenotypic traits for LR, RF, and SVM. For each of the NN models, all the features were used for inferring, and the resulting analysis with SHapley Additive exPlanations (SHAP) [51], which was performed in order to indicate the contribution of each feature to the model’s output, did not reveal any specific gene with significant importance over others. To enable the retrieval of genes for all the classifiers, the second-best model among the remaining ones was utilized for functions whose model was trained using NN.

Among the 11,469 KOs present in the dataset, 11,266 were identified as important for at least one function. The number of genes per function exhibited considerable variation (Table S3). The range extended from 0 relevant genes for the ineffective “dark sulfite oxidation” model, where all algorithms but NN scored 0, to over 11,000 (Figure S7B, S7C, S7D). These counts displayed a strong relationship with the used algorithms, with SVM models having the highest average number of inferred genes, and LR exhibiting relevance for either a remarkably small (l1 logistic regression) or extremely large (l2 logistic regression) number of KOs. Analysis of the classifier’s performances revealed that models with both low and high amounts of retrieved genes showed high classification performance (MCC > 0.7). Poorly performing classifiers can exhibit both a small number of inferred genes (e.g. 0 genes for “dark sulfite oxidation”) or a high number (e.g. 11,267 for “anoxygenic photoautotrophy Fe oxidizing”). Notably, most models utilized a high number of genes, including “fermentation” using 7,358 genes, and “mammal gut” employing 11,309, for an average of around 7,000. Only three classifiers - “methanogenesis”, “arsenite oxidation energy yielding” and “fish parasites” - achieved accurate performance while using fewer than 100 genes (37, 26, and 66 respectively).

No correlation was present between the number of detected genes and the number of genomes in the corresponding functional class (Spearman’s correlation coefficient ≈ 0.07, *p*-value = 0.51, Figure S7C).

Analysis of the retrieved genes confirmed that most of the classes were associated with KOs with low feature importance rather than a few more relevant ones (Table S3), as confirmed by the strong negative correlation between the number of positively associated genes and the average value of the importance score (Spearman’s correlation coefficient ≈ -0.61, *p*-value = 2.26 × 10^− 10^). For instance, over 350 KOs were identified as important for predicting the “methanogenesis by disproportionation of methyl groups” class (MCC of 1 in the test set), in contrast with the 37 genes associated with the broader “methanogenesis” class. While some of the identified genes were already recognized to be involved in the process, such as the *mcrC* gene, others selected by the classifier were not associated with the corresponding KEGG modules, but were present in related pathways such as “coenzyme biosynthesis”.

### Validation on independent genomes

An additional set of 4,146 genomes, which were not part of the initial training set, but belonged to taxa stored in FAPROTAX, was gathered along with their corresponding functions. These real genome-function associations were treated as true positives, enabling their use in evaluating the performance of the tool. A total of 11,195 functions were assigned across the entire dataset, with chemoheterotrophy being the most abundant, associated with over 3,000 organisms. The analysis revealed that only 67 out of the 89 functions were performed by at least one of the organisms in the dataset. For all these classes with associated genomes, the MCC score was calculated to measure the performance (Figure S8, Table S2).

In general, the tool performance was lower than in the test set: only 20 classifiers had MCC > 0.7, and 29 had MCC < 0.4. However, the performance efficiency might have been affected by the small number of true positives associated with some functions. Indeed, of the 67 functions carried out in the validation dataset, 13 were performed by single genomes. The low number of true positives in the validation set associated with some functions, independently from the number of genomes per functional class used in the training, was not considered by the evaluation metric and also penalized the obtained results (Table S2). Because the MCC score highly values the ability to predict true positives, if a classifier does not succeed, the score drops independently from how small their number might be. For instance, the “arsenate detoxification” classifier, despite showing high performance in the test set, received an MCC of 0 due to the lack of prediction of the single organism performing the function. A similar result was found for the “hydrogenotrophic methanogenesis” class: despite successfully predicting the single actual hydrogenotrophic methanogen, it obtained a low score due to also predicting 9 false positives out of 4,146.

It was not possible to use MCC to evaluate the performance of the tool for the remaining 22 classes, since this metric is equal to 0 when no true positive samples are present in the dataset. Thus, confusion matrices were computed to investigate the efficiency of the tool (Table S2). With this approach, a perfect classification was achieved by 4 of these classifiers, while the other 18 performed fairly well. Some false positives were predicted, but their number was very low if compared with the true negatives.

**Fig. 3:**
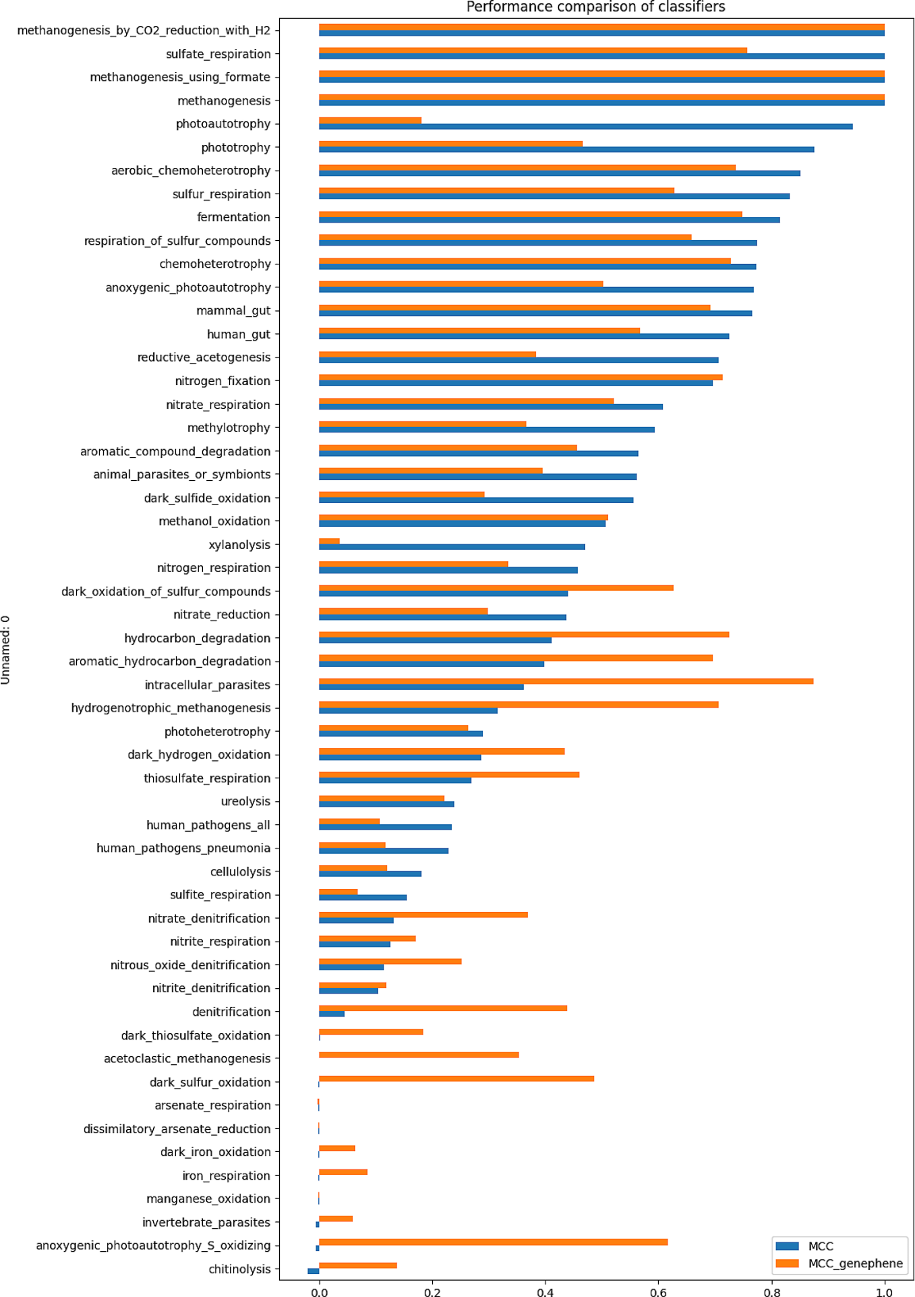
Comparison of MCC scores obtained in the described validation set by MICROPHERRET and by GenePhene

The performance of MICROPHERRET was compared with GenePhene, a machine learning tool previously developed [34]. The latter could predict organisms’ ability to perform 84 functions, 83 of which were shared with the newly developed tool. By using these predictions, MCC was computed for all the classes with at least one true positive, which were 63 when considering those in common between the tools. Among these 63 classes, MICROPHERRET outperformed GenePhene in 63% of the cases, matched performance in 8%, and was outperformed in 29% (Fig. 3, Table S6). In three cases – “methanogenesis”, “methanogenesis using formate” and “methanogenesis by CO_2_ reduction with H_2_” – both tools correctly predicted all the species with no false positives. Regarding the 20 classes without true positives for GenePhene, MICROPHERRET performed better in 19 cases. GenePhene typically produces a higher number of false positives compared to MICROPHERRET (14,206 for GenePhene vs. 6,156 for MICROPHERRET, Table S6), resulting in a higher false positive rate for 18 classes (22%). Notably, 3,052 false positives predicted by GenePhene were assigned to classes where no true positives were expected, highlighting its lower specificity.

### Validation on simulated fragmented genomes

**Fig. 4:**
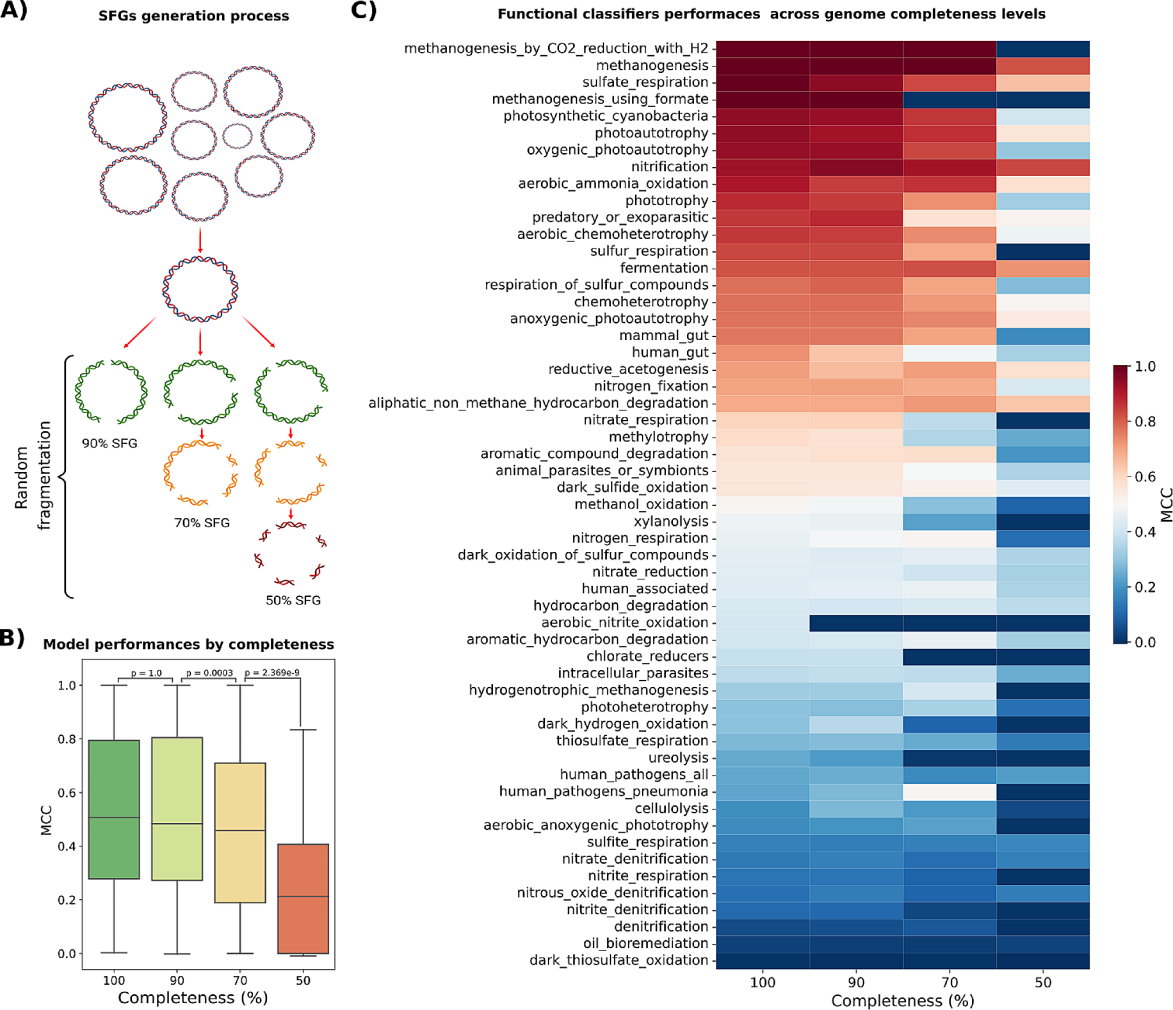
(**A**) Generation of simulated fragmented genomes (SFG) at different completeness levels through randomic genome fragmentation. (**B**) Classification performance distributions on complete genomes and at the different SFG completeness levels, with *p*-values from Wilcoxon tests expressing performance differences across groups. (**C**) Comparison of functional classifiers performance on complete genomes and SFGs at different completeness levels

The randomic removal of genomic regions from each genome present in the initial set of 4,146 genomes was performed to assess the performance of MICROPHERRET on partial genomes simulating the characteristics of MAGs. This process resulted in the generation of a dataset of simulated fragmented genomes (SFGs). Three distinct completeness levels were simulated, namely 90%, 70%, and 50% (Fig. 4A) (see Methods). The resulting SFGs underwent gene finding and annotation with the same tools previously selected for the complete genomes. The outcomes were compared with the 67 classifiers previously obtained for the original dataset.

As expected, the performance of classifiers in the 90% SFG decreased in comparison to the original dataset (Fig. 4B-C) More specifically, 25 out of 67 classifiers evidenced a decreased performance, and notably, the “aerobic nitrite oxidation” class demonstrated a substantial decline of 40% in classification power (Table S2). This could be attributed to the low number of genomes in this class that can determine a strong impact of a single misclassification on the MCC calculation. For the remaining 24 classifiers, 11 exhibited a decrease between 9% and 1%, while for others, the change was less than 1%. More surprisingly, 31 classifiers demonstrated increased MCC values, although for 18 of them by less than 1%, and for the other 13 the increase ranged between 1% and 9%. The remaining classifiers showed no change.

Comparable trends were observed for genomes fragmented to a 70% level, with 38 classifiers performing better with complete genomes, 6 exhibiting no difference, and 23 demonstrating better performance with incomplete genomes. However, for genomes fragmented at 50%, the overall performance of all classifiers was consistently lower across all the functions. Surprisingly, classes describing certain functions, such as methanogenesis and nitrification, maintained high MCC values. While certain classifiers displayed improved performance, they pertained to classes with low MCC values even for the complete genomes. In all other instances, the performance was suboptimal, with only two classifiers achieving an MCC higher than 0.7.

A Wilcoxon test was employed to assess potential variations in the performance of all classifiers across distinct levels of genome fragmentation. Results indicated no statistically significant differences between the performance of classifiers on complete genomes and those fragmented at 90%. However, a significant divergence in performance was observed between 90% and 70% completeness, as well as between 70% and 50% completeness. Concurrently, Pearson’s correlation test was conducted to examine the association between the MCC values of classifiers tested on complete genomes and their fragmented counterparts. The analysis revealed a high degree of correlation between complete genomes and those fragmented at 90% and 70% completeness, with correlation coefficients of 0.99 and 0.91, respectively. In contrast, the correlation was comparatively lower for the 50% completeness scenario, yielding a correlation coefficient of 0.76.

### Application to the Biogas Microbiome database

In order to evaluate the performance of MICROPHERRET in a real-case scenario, it was tested on a dataset with well-known functional characteristics that allow the comparison between the actual data and the predictions. Thus, the tool was applied to the Biogas Microbiome database [52], comprising 4,568 MAGs found in anaerobic digesters of four different continents [53]. In contrast to the genome sets utilized above, these MAGs presented a certain level of contamination, albeit generally low, with a maximum of 10%. This introduced a source of confounding for the generated models, whose robustness to contamination noise could thereby be tested.

MAG taxonomy was utilized to cross-reference them against the FAPROTAX database, resulting in 635 MAGs being successfully identified as associated with 74 functions. Their completeness was assessed and 69 MAGs were between 50% and 70% complete, 213 between 70% and 90% complete and the remaining 355 had completeness over 90% (Table S7). The average MCC value is 0.66 for the first group (completeness < 70%), while it increases to over 0.74 for the other groups. The established MAG-function links were employed as “true labels” for comparison and evaluation, wherein MICROPHERRET predicted the functions of these 635 MAGs. MCC was computed for the 74 classes exhibiting any true positives in the dataset (Table S4). Out of the 74 classes, 61 classifiers demonstrated satisfactory performance (MCC > 0.4), with 19 models exhibiting high classification efficiency (MCC > 0.7). Notably, models specific to methanogenesis, except “methanogenesis using formate,” performed exceptionally well, including the “hydrogenotrophic methanogenesis” class, which had previously shown poor performance in the validation set. The remaining 15 classifiers, without corresponding functions in the dataset, exhibited some false positive and negative classifications. Among these, 12 functions were correctly not predicted from any MAGs, while three models produced only a limited number of false positives, reaching a maximum of 0.8% for “aliphatic non-methane hydrocarbon degradation (Table S4).

**Fig. 5:**
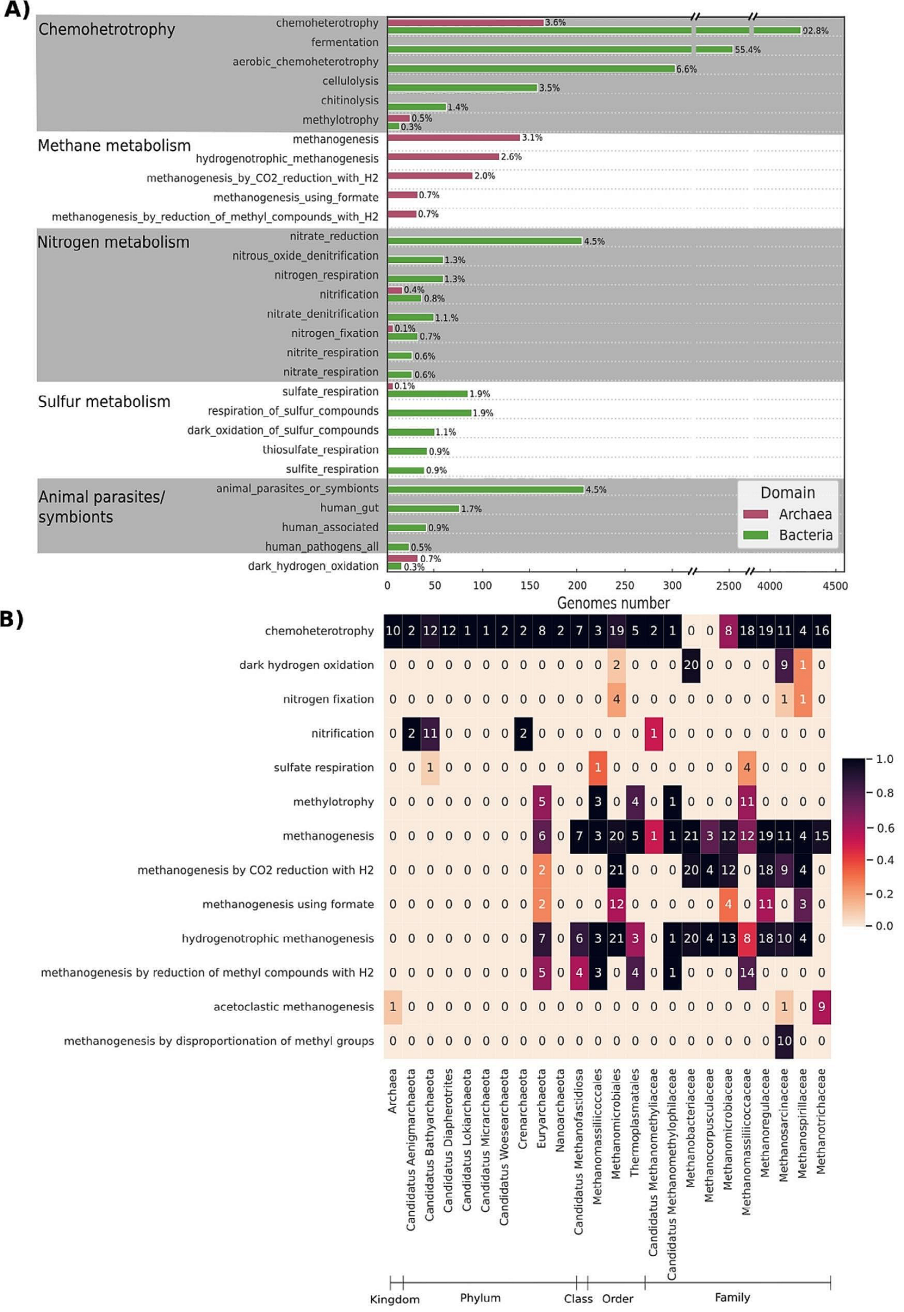
(**A**) The percentage of MAGs present in the Biogas Microbiome database that were associated with specific functions is displayed. For visualization purposes, only functions exceeding 0.5% of genomes are reported. (**B**) Distribution of the performance of the 18 functions predicted for archaeal MAGs in the anaerobic digestion dataset across the archaeal taxonomic groups. The organisms were grouped at family level or higher, according to the available taxonomy. The values were normalized to the number of genomes in each rank and the displayed color scale represents the proportion of genomes associated with the functions per taxonomic rank. The number of genomes associated with the functions is indicated in each cell

Following the evaluation of this subset of 635 MAGs, the tool was employed to predict phenotypes associated with the complete set in the Biogas Microbiome database. In total, 9,468 functions were predicted. Figure 5A shows the percentage of MAGs performing functions exhibited by over 0.5% of the genomes.

The subsequent analysis focused on the 198 detected archaeal MAGs, aiming to more closely verify the agreement of model predictions with previous characterization of low-level taxa. Among these, 153 genomes were associated with methanogenesis, with 140 predicted to belong to the higher-level “methanogenesis” class. Further examination of the remaining 13 MAGs, linked to methanogenesis subclasses but not to the main class, revealed that these genomes were missing at least half of the 37 relevant genes detected by the model. The identified methanogens were predominantly within the Euryarchaeota phylum, which is well known for including organisms involved in methane metabolism [55, 56].

The analysis also identified 46 archaea not linked to any form of methanogenesis, primarily classified at the phylum level. These were mostly non-Euryarchaeota genomes - such as the 13 Candidatus Batyarchaeota and the 12 Candidatus Diapherotrites MAGs - and were all correctly classified as non-methanogens due to their lack of relevant genes (Table S8). Specifically, 42 MAGs lacked copy numbers of the 10 most critical KEGG Orthologs (KOs) for the function, reinforcing their correct classification as non-methanogens. In total, the archaeal MAGs were identified as associated with 18 functions (Fig. 5B). As anticipated, the predictions aligned with existing knowledge, with methanogenesis exclusively linked to taxa within the Euryarchaeota phylum [55, 57].

### New acetoclastic methanogenesis classifier

A curated version of the “acetoclastic methanogenesis” class was formulated to investigate whether the inclusion of more genomes exhibiting this specific function could enhance the classification performance. This curation also served as a benchmark for testing the incorporation of new user-defined classes.

In the FAPROTAX database, the “acetoclastic methanogenesis” group originally comprised four *Methanosarcina* species and the Methanosaetaceae family. However, it is established that additional members within the *Methanosarcina* genus can also perform methanogenesis from acetate [58, 59]. The revised class included a greater number of genomes, encompassing 11 from the *Methanosarcina* genus, 3 from *Methanothrix*, and a single genome belonging to the *Methanocalculus alkaliphilus* species [60]. 17 additional strains from *Methanosarcina* and *Methanosaeta* were reserved for validation purposes. Throughout the model selection process, it was determined that the SVM with a linear kernel exhibited superior classification compared to LR, which was the previously identified best model. Training the classifier with the updated genomes resulted in enhanced performance across all previously tested datasets (Figure S9). Initially, the model underwent validation on a dataset comprising the aforementioned 17 acetoclastic methanogens and 100 genomes associated with alternative functions, including hydrogenotrophic methanogens.

The new model showcased a greater improvement compared to the original acetoclastic classifier (0.45 MCC previously, now 0.86). Out of the 17 acetoclastic methanogens, the new model accurately predicted 13 without any false positives, while the previous model only correctly identified 5 and incorrectly classified a hydrogenotrophic methanogen as acetoclastic. Notably, the new model achieved perfect predictions in the separate dataset of 4,146 genomes from the FAPROTAX database (MCC = 1), accurately classifying the two existing acetoclastic methanogens. In stark contrast, the old classifier failed to classify any of them. Furthermore, this new model outperformed the “acetoclastic methanogenesis” model in GenePhene (MCC = 0.35). Substantial improvements were also observed in the anaerobic digestions MAGs dataset. The new model successfully predicted 19 members of the Methanosarcinaceae and Methanotrichaceae families as acetoclastic methanogens. Additionally, two MAGs previously misclassified were now correctly assigned as non-acetoclastic methanogens.

## Discussion

This study implemented a machine learning approach that integrated various supervised learning algorithms to predict microbial functions based on their genomic content. Inspired by previous work [34], the implemented strategy involved training and optimizing 89 function-specific binary classifiers using a dataset extracted from the FAPROTAX database, leveraging KEGG Orthologs as the genetic information source. KO coverage varied across the training set, but the average coverage was consistent with literature values [61]. While Pfam entries covered 90 ± 3% of annotated genes, domain-based annotation methods like Pfam might lead to not realistic annotation coverage because the presence of a domain in a predicted protein sequence may not be sufficient to assign function, requiring more stringent annotation methods. KOs were specifically chosen for this study since their orthology-based origin allows us to exploit knowledge of model organisms to infer information in less-studied ones. Additionally, KEGG’s comprehensiveness facilitates the contextualization of genes in more general networks. GenePhene developers tested for the best gene annotation for the classification comparing KEGG and Pfam, obtaining a better average performance using KOs, further supporting the validity of our decision. Analysis of the KOs coverage across functional classes proved that KO coverage does not affect the models performance. However, integration of different gene annotations might increase the performance efficiency.

Notably, our program demonstrates enhanced accuracy and efficacy in functional predictions when compared with GenePhene. Several differences emerged when comparing the two tools. First, employing FAPROTAX as a source for both functional and genomic information resulted in a larger number of genomes in the training set in this study, with over 14,000 genomes against 9,000 of GenePhene. This allowed to increase the classification potential by expanding the number of positive and negative samples in all classes. Additionally, the incorporation of multiple supervised learning methods within this strategy, as opposed to the training of only LR models in GenePhene, boosted the prediction accuracy and enhanced the extraction of functional information. This helped to capture hidden relationships between certain functions and genes previously not found, as in the methanogenesis case. Moreover, the integration of optimization techniques in the present approach aimed to obtain high quality level models, further increasing the prediction accuracy. MICROPHERRET also allows the classification of MAG functions even with incomplete genomes, as seen in the validation of the Biogas Microbiome database. The tool also has the capability of generating new classifiers with tailored functions, allowing customization to both existing and unknown classes by the users.

A total of 81 models with excellent performance and 5 models with good performance were obtained. There was not a single machine learning method that showed overall a better performance over others. High variance in the performance of some classifiers was seen across validation and tuning sets during training. For example, the “aerobic nitrite oxidation” classifier exhibited high performance in the test set but lower performance in the validation set, indicating potential generalization instability. To address this, future efforts may focus on targeted optimization strategies tailored to improve the robustness and generalization capabilities. This may involve expanding the number of available genomes, as shown in the case of the acetoclastic methanogenesis classifier. The high number of features used for the prediction (over 10,000 KOs) affected the classification power of NNs. Grouping features, for example by clustering together KOs in their respective pathways, could increase NN’s classification ability while also balancing the need to properly discern more general classes that are not linked to specific pathways, such as “oil bioremediation”.

The high number of phenotypes for which efficient models were trained represents a unique and significant advantage. Exploiting the structure of the FAPROTAX database, MICROPHERRET proved to be able to classify microorganisms according to a diverse range of features, spanning from many specific metabolic capabilities to the ecological niche they belong to. This diversity constitutes a unique aspect of this tool, which presents itself as a classifier of a wide range of phenotypes and is opposed to several more specific projects. For instance, the Diaspora project [62] uses the information stored in the BacDive [17] database to train SVM models for critical phenotypes for cultivating conditions, obtaining a small number of efficient models.

Importantly, several predicted functions such as the “gut” and “pathogens related” ones are not directly associated with specific KEGG pathways or modules. This stands as a significant advantage since it allows the prediction of phenotypes and associated genes for which no module or pathway structure is available in KEGG. This flexibility expands the tool’s potential applications, as it is not constrained by the limitations of KEGG’s structure and availability, relying instead on KEGG ortholog codes only as a source of features for prediction. The same consideration can also be made in the case of METABOLIC [16] and KEMET [15], where the predictions of the functional roles of microbial genomes are based on a series of different databases but are ultimately mapped to KEGG pathways.

While using the FAPROTAX database as a base for MICROPHERRET design led to the mentioned important advantages, modifying or broadening some functional classes could potentially address the previously described generalizability issue and increase the prediction efficiency. For example, the class “anoxygenic photoautotrophy Fe oxidizing”, was characterized by a limited set of genomes, several of which shared with the classes “anoxygenic photoautotrophy S oxidizing” and “anoxygenic photoautotrophy H_2_ oxidizing”. This structure might have prevented the detection of relevant genetic content to distinguish it from these similar classes, decreasing the model’s accuracy. On the other hand, the more general class “anoxygenic photoautotrophy”, which includes all the ones previously mentioned, displayed a better classification performance. This suggests that integrating specific classes in a broader one might have the potential of augmenting the classification power.

To address the need for modifying the composition of functional classes in FAPROTAX and, as an example for the creation of new classes, the acetoclastic methanogenesis was refined. This new classifier not only showed significant improvements over the one previously generated in this study but also outperformed GenePhene. The results underscored the significant progress achievable through the optimization of the initial function, highlighting the critical role of class composition in performance efficiency and proving the validity of the functional prediction approach proposed in this work. Importantly, the outlined pipeline provides a framework that can be extended to entirely new classes by incorporating genomes of organisms performing functions absent in FAPROTAX. This flexible approach empowers users to generate new classes of organisms for predicting functions of interest within the conceptual framework described, showcasing the vast versatility of this methodology. Moreover, this method allows the expansion of MICROPHERRET genome and function database upon the inclusion of novel information in future updates of FAPROTAX.

The identification of the most crucial genes involved in the classification of all the models allowed to investigate significative genotype-phenotype associations. While not all the detected signals are believed to be relevant, genes with the highest rankings identified by accurate models should be considered potentially informative for assessing an organism’s ability to execute a specific function. For example, several of the 100 most important genes for fermentation belonged to KEGG modules involved in the degradation of carbohydrates, lipids, and protein compounds. Moreover, two genes involved in ribonucleotide reduction (*nrdD* and *nrdG*), acting exclusively in anaerobic conditions [63], underscored and emphasized on the accuracy of the models. These genes play a vital role in synthesizing deoxyribonucleotides required for DNA synthesis and have been demonstrated as essential for the growth of various fermentative organisms under anaerobic conditions [64, 65]. Conversely, classifiers with lower performance efficiency associated with high numbers of KOs likely failed to capture significant connections beyond taxonomic ones, such as those specific for arsenate and manganese respiration with more than 11,000 relevant genes and MCC of 0.47 and 0.55.

No significant correlation was observed between the number of detected genes and the number of genomes in the functional classes. Analogously, there was no correlation between the number of genomes and the performance of the classifier. These aspects, together with the efficiency obtained by models for more in-depth classes, highlight the importance of having annotated genes coherently associated with the function, stressing the relevance of the functional class composition, rather than the number of genomes in the class. Consequently, the low performance accuracy of the “oil bioremediation” model might be attributed to the class structure, marked by organisms that differ significantly from each other or lack a particular set of common genes. This might have hindered the identification of common relevant genetic content, affecting the classification. On the other hand, the training set for the “acetoclastic methanogenesis” class only had 15 associated genomes, four less than “oil bioremediation”, underscoring that the presence of specific genes is a more pivotal factor than the number of genomes used for classification.

While the complex genotype-phenotype relationships and the vast differences between the analyzed phenotypes make it difficult to define the structure of the ideal MICROPHERRET functional class, the discussed results have allowed us to outline conditions that enhance the efficiency of MICROPHERRET’s models, providing useful guidelines for its usage. The functions predicted with the highest accuracy are those with a well-defined set of genes, such as methanogenesis, as expected: the stronger the relationships between the presence of specific genes and the function, the easier it should be for the algorithms to model them.

The genomes from which the models extract information for prediction (i.e., those that form the functional class) are crucial. As demonstrated by acetoclastic methanogenesis, a high number of genomes is not necessary for excellent performance. However, more complex functions spread throughout the phylogenetic tree, such as fermentation, require a higher number of genomes to facilitate the learning process. Therefore, we advise to provide as many genomes as possible for the functional class. A useful approach might be to begin with broader functions of interest and then refine the focus. Indeed, as previously stated, integrating specific classes into a more general one might enhance the classification. For example, volatile fatty acid production could be implemented as a new class that incorporates all the possible pathways for the production, similarly to the “methanogenesis” class which is comprehensive of all the subclasses. Alternatively, if a more precise classification for one or more volatile fatty acids is needed, it is also possible to create separate classes for each specific function, provided that a sufficient number of genomes associated to the functions is available. These genomes must satisfy specific characteristics. First, if the target class is known to be associated with specific genes, these genes should be present within the provided genomes. Moreover, the genomes should come from taxonomically diverse sources, minimizing redundancy from closely related species, and should be of high quality (in our training set, we used completeness > 90% and contamination < 5%) to ensure correct genotype-phenotype associations.

Conversely, if users wish to use the already available models in MICROPHERRET without customizing the approach, the only requirement is to provide MAGs of the highest possible quality, aiming for completeness > 70%, the proven threshold for MICROPHERRET’s general efficiency. However, some function-specific models in the tool may not fully align with the recommended guidelines and might not achieve optimal performance. For example, analysis of the tool performance across broader functional groups, i.e. superclasses, showed that while MICROPHERRET can predict most of the analysed metabolisms, function-specific models associated with sulfur metabolism and phototrophy have a lower performance. Additionally, as shown for the hydrogenotrophic methanogenesis model in the validation, if the dataset that the user wishes to analyze is highly imbalanced and a function is scarcely present, the model’s performance might decrease.

The provided guidelines not only offer a more comprehensive understanding of the explained results and reiterate which trained models are highly efficient, but also give clear instructions on the characteristics that a new or refined class should possess to have a higher probability of being efficient. This aids users in their specific analyses and helps expand the tool’s application.

Further comparisons with the GenePhene models revealed differences in the number of retrieved genes and their categorization, particularly in the KEGG methanogenesis pathway. However, these differences did not detrimentally impact classifier performance, and the achievement of perfect evaluation scores in both analyses suggested the successful applicability of both models for methanogenesis prediction from gene content.

These notable disparities between the obtained lists of genes and the corresponding KEGG modules implied that genes not organized in modules or pathways could still be valuable in predicting the ability of a microbial species to perform a specific function. The confirmation of model success in predicting methanogenesis from gene content, despite differences in identified genes from established databases, highlights the potential of these models to uncover novel genetic associations not explicitly captured by existing knowledge frameworks.

Overall, the machine learning-based functional association exhibited the capability to transcend the structured modules and pathways of existing databases, representing a notable advantage over traditional methods.

The validation of the models in this study, conducted on independent datasets and simulated fragmented genomes, underscored their effectiveness in establishing functional associations across both complete and incomplete genomes. Depending on the level of completeness, MAGs may not possess all the necessary genes for performing the entire process described in a pathway, highlighting the challenge of predicting functions with a limited number of relevant genes with traditional methods, such as KEGG annotations alone. Furthermore, fragmented genomes are characterized by a generalized low copy number of the other pertinent genes. MICROPHERRET successfully achieved a reasonably robust classification for SFGs with completeness over 70%, whereas for values lower than this threshold, acquiring sufficient information for the machine learning algorithms to operate effectively becomes challenging. This remarkable achievement in functionally classifying lower-quality genomes validates the tool’s proficiency in handling MAGs and could be partly attributed to the high number of genes deemed as relevant by the majority of classifiers. Indeed, the potential absence of a relevant gene due to poor genome quality is less likely to impact the classification since numerous other genes have been used by the models. This aspect represents a significant advantage compared to other functional annotation strategies, like metabolic flux balance, where gap-filling algorithms may struggle to solve the incomplete structure of certain reaction networks, precluding the modeling [66]. Additionally, MICROPHERRET stands out from annotation methods relying on KEGG pathway completeness, where the absence of a few genes can hinder accurate classification. For instance, in a previous study [52], 2 out of the 7 MAGs belonging to the Methanomicrobiales order - organisms recognized for their capability to perform hydrogenotrophic methanogenesis [56] - were not considered as able to produce methane from CO_2_ because characterized by an incomplete hydrogenotrophic methanogenesis KEGG module (M00567). The same situation arose with one of the 5 *Methanothermobacter* genomes, acknowledged as hydrogenotrophic methanogens commonly found in high-temperature anaerobic environments [67].

Notably, in the case of the SFGs, there were classifiers capable of outperforming the ones tested on complete genomes for the 90% SFGs. This improved performance could be due to the copy number reduction or the complete absence of some KOs, potentially aiding in mitigating confounding factors during classification. Another possible explanation could be found in the size of the functional classes. Indeed, as stated above, classes with few genomes have a high variability in their classification scores which can be dramatically affected by a single misclassification. The presence of contamination within MAGs could also potentially hinder the classifier’s ability to predict functions correctly. While a careful assessment of contamination should be carried out before annotation, genes incorrectly assigned to the MAG could hamper the classification process and introduce errors in the correct assignment of functions.

MICROPHERRET successfully predicted functions for MAGs within an anaerobic digestion environment, yielding results consistent with existing literature. The “chemoheterotrophy” class, given its high species count, was linked to the majority of MAGs. This might be expected since chemoheterotrophy is a generic metabolic strategy exhibited by many microorganisms [68]. Additionally, fermentation was abundantly predicted, aligning with its prevalence in anaerobic digestion environments [69]. All forms of methanogenesis were identified by the tool, albeit some, such as methylotrophic and acetoclastic methanogenesis, in relatively lower quantities. This overall limited presence of organisms associated with methanogenesis confirmed previous findings, as an anaerobic digestion microbiome has a funnel-shaped organization where the methanogenesis at the last step is performed by a limited number of specialized archaea [70]. The obtained results further showed the applicability of MICROPHERRET in a real-case scenario.

## Conclusions

The categorization of prokaryotic genomes based on functionality remains a significant challenge in genomic analysis, primarily reliant on manual associations and limited to isolated microbes. This study explores the potential of machine learning as a systematic approach to infer the functions of unknown genomes by leveraging information derived from annotated genes. This novel process not only enables the elucidation of concealed or potential new mechanisms in species phylogenetically close to those with well-established roles but in principle also for completely uncharacterized ones. This investigation lays a foundation for a robust conceptual framework that proficiently combines supervised learning methods for the functional classification of microorganisms. Combining different annotation strategies with KEGG, such as EC or Pfam [71] might further increase the information available for the training.

The resultant tool stands as a valuable resource for predicting the 86 trained functions from FAPROTAX. Furthermore, MICROPHERRET user-friendly design facilitates straightforward curation by users, allowing for the classification of organisms according to specific functions of interest. The overarching aim is to enhance the efficiency and accessibility of functional classification, fostering advancements in understanding microbial genomics.

### Electronic supplementary material

Below is the link to the electronic supplementary material.

Supplementary Material 1

Supplementary Material 2

## Data Availability

MICROPHERRET and all the reproducible code used in this paper are available on https://github.com/BizzoTL/MICROPHERRET/.
