# Supplementary material for: MICROPHERRET: MICRObial PHEnotypic tRait ClassifieR using Machine lEarning Techniques
Source: Environ Microbiome. 2024 Aug 8;19:58. doi: 10.1186/s40793-024-00600-6 (PMC11308548; doi:10.1186/s40793-024-00600-6)
Supplement: Supplementary file 2 — Supplementary Material 2 [file 40793_2024_600_MOESM2_ESM.docx]

**SUPPLEMENTARY FIGURES AND TABLES**


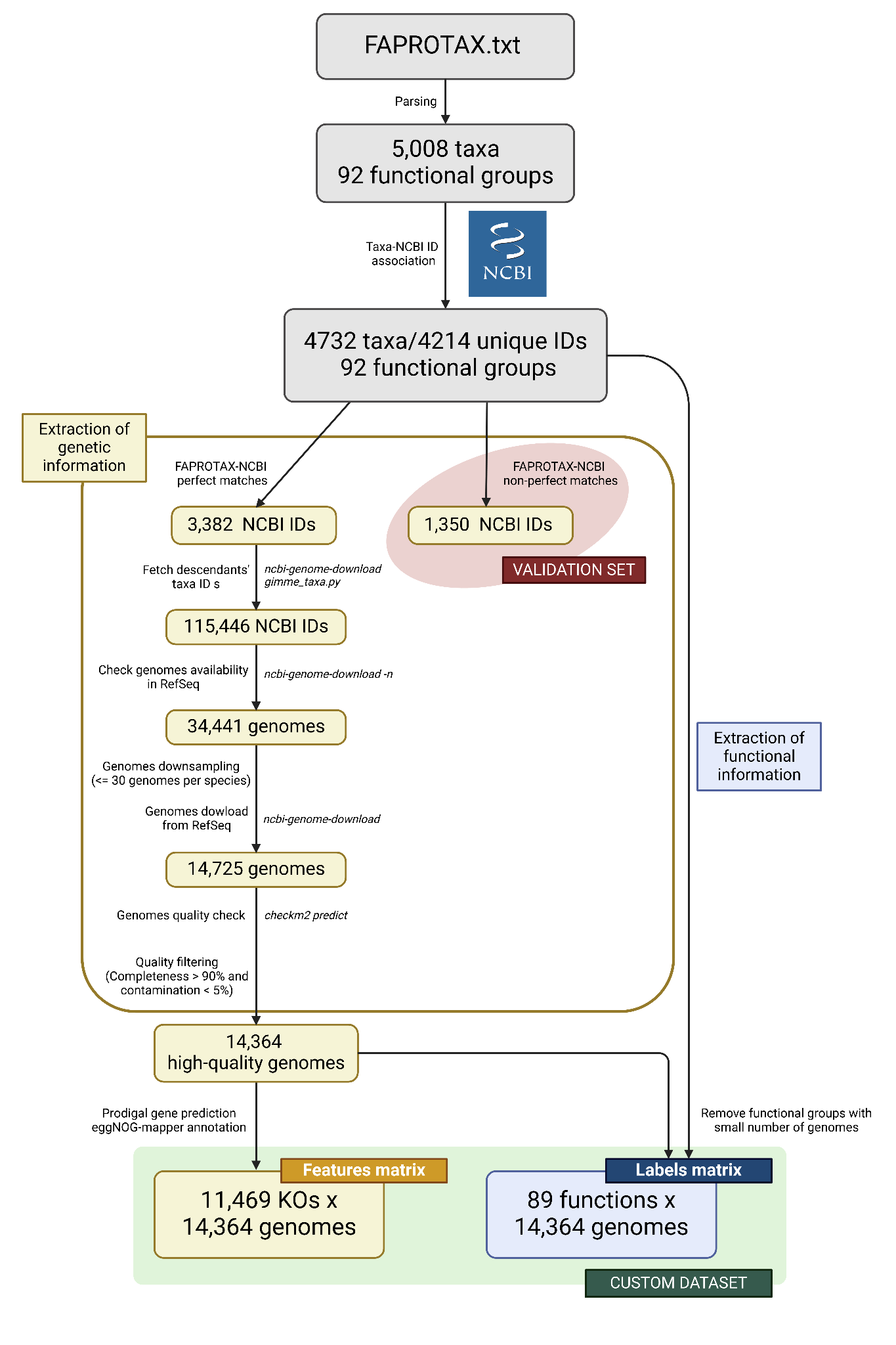


*Supplementary Figure S1.* Comprehensive representation of dataset generation to extract information from FAPROTAX database.


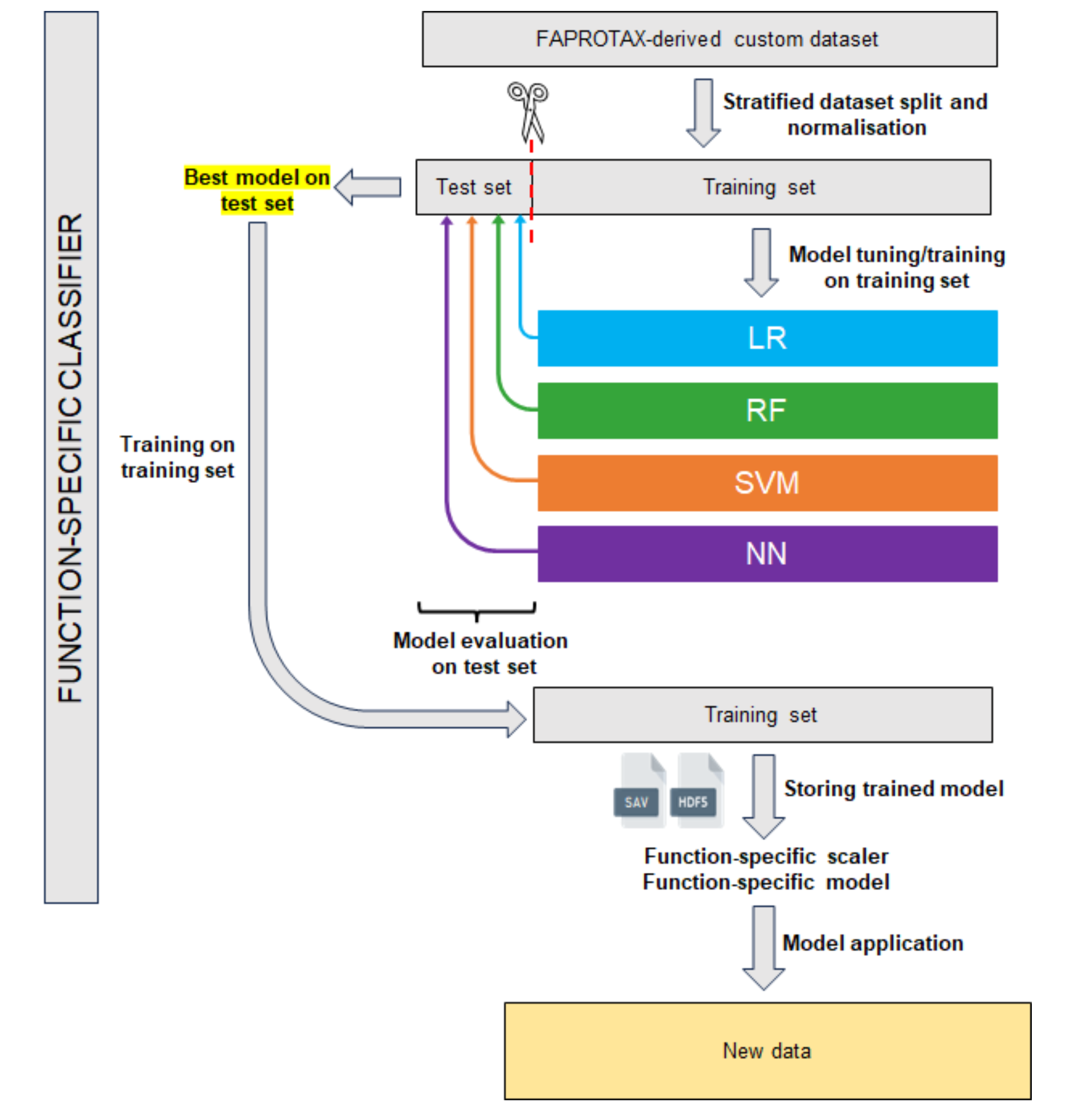


*Supplementary Figure S.2.* Function-specific classifiers training strategy. For each function, the dataset was split into training and test sets in a stratified manner and normalisation was performed. Logistic regression (LR), random forest (RF), support vector machines (SVM) and neural networks (NN) were optimised and trained on the training set. The models were evaluated on the test set and their performances were compared. The resulting most efficient model was trained on the training set and stored in .sav or .hdf5 files: the “scaler” file contained information on the normalisation step, while the “model” file stored the classifier itself. The saved files can be used to apply the obtained model to new datasets.


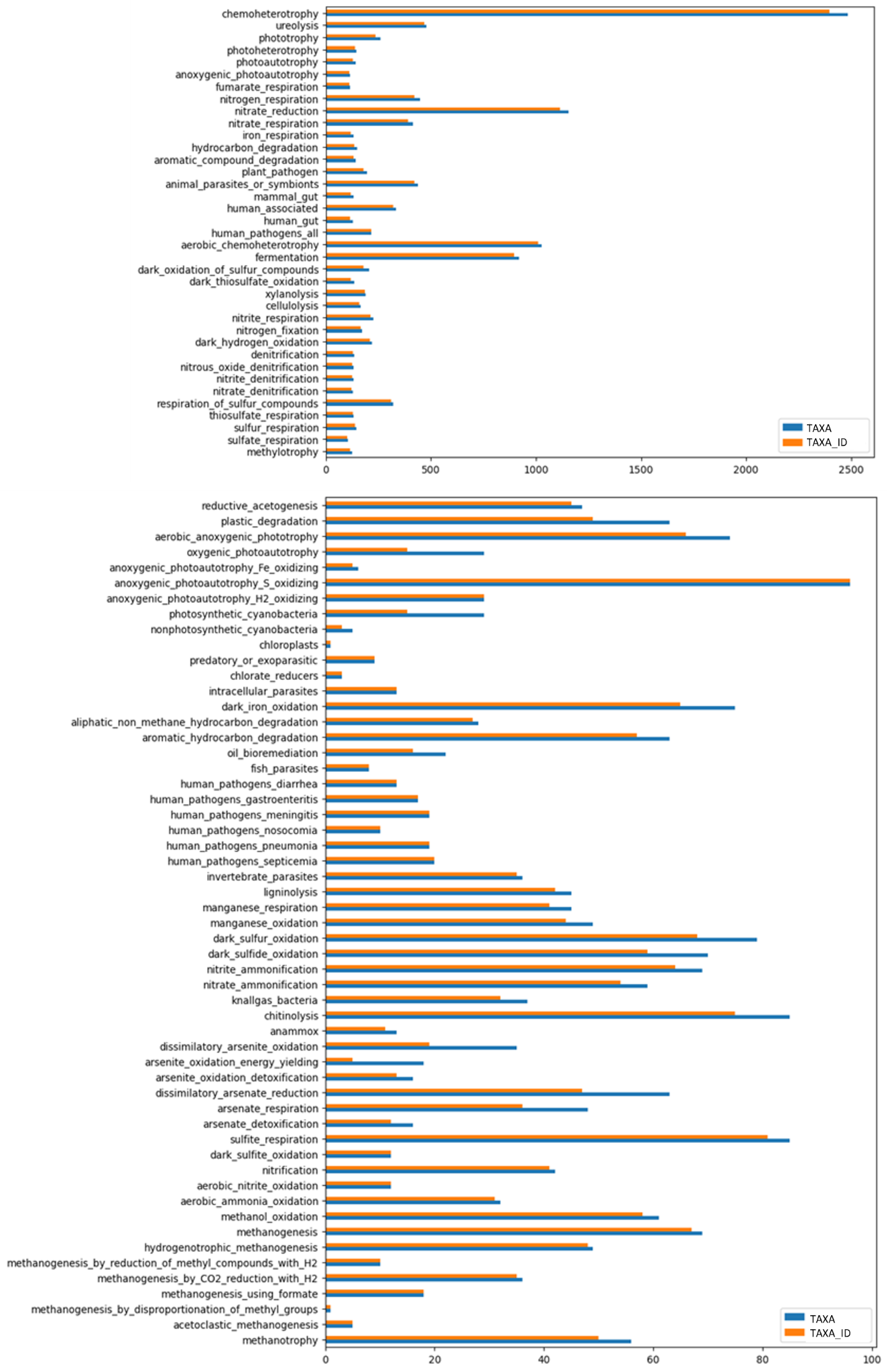


*Supplementary Figure S.3*. Number of taxonomic entries (TAXA) and number of taxonomic entries for which the corresponding NCBI IDs (TAXA_ID) were detected per FAPROTAX functional group.


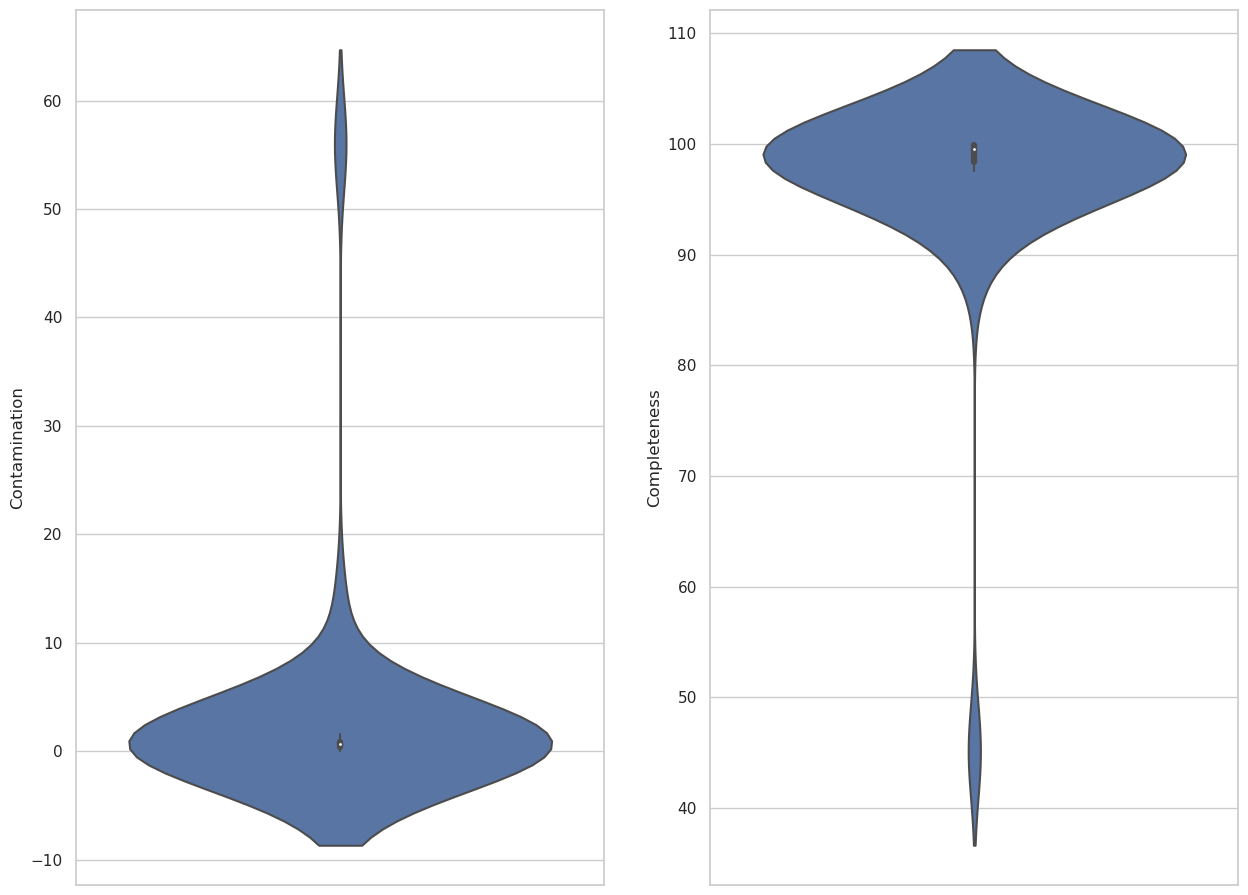


*Supplementary Figure S.4*. Distribution of the contamination and completeness of the 14,725 genomes downloaded from the NCBI database.


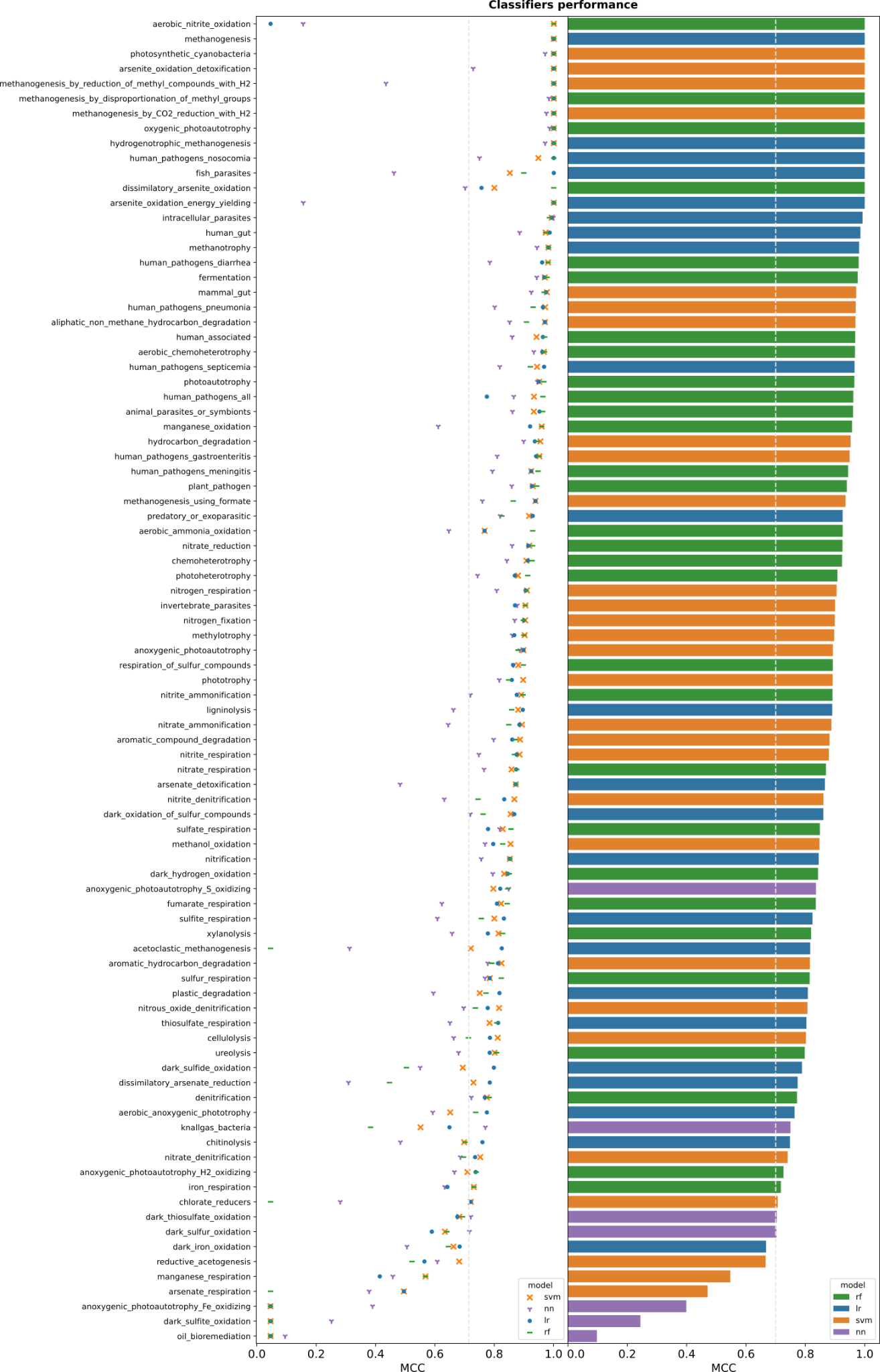


*Supplementary figure S.5.* MCC scores on the test sets of the four applied algorithms and MCC score of the selected best algorithm per functional class.


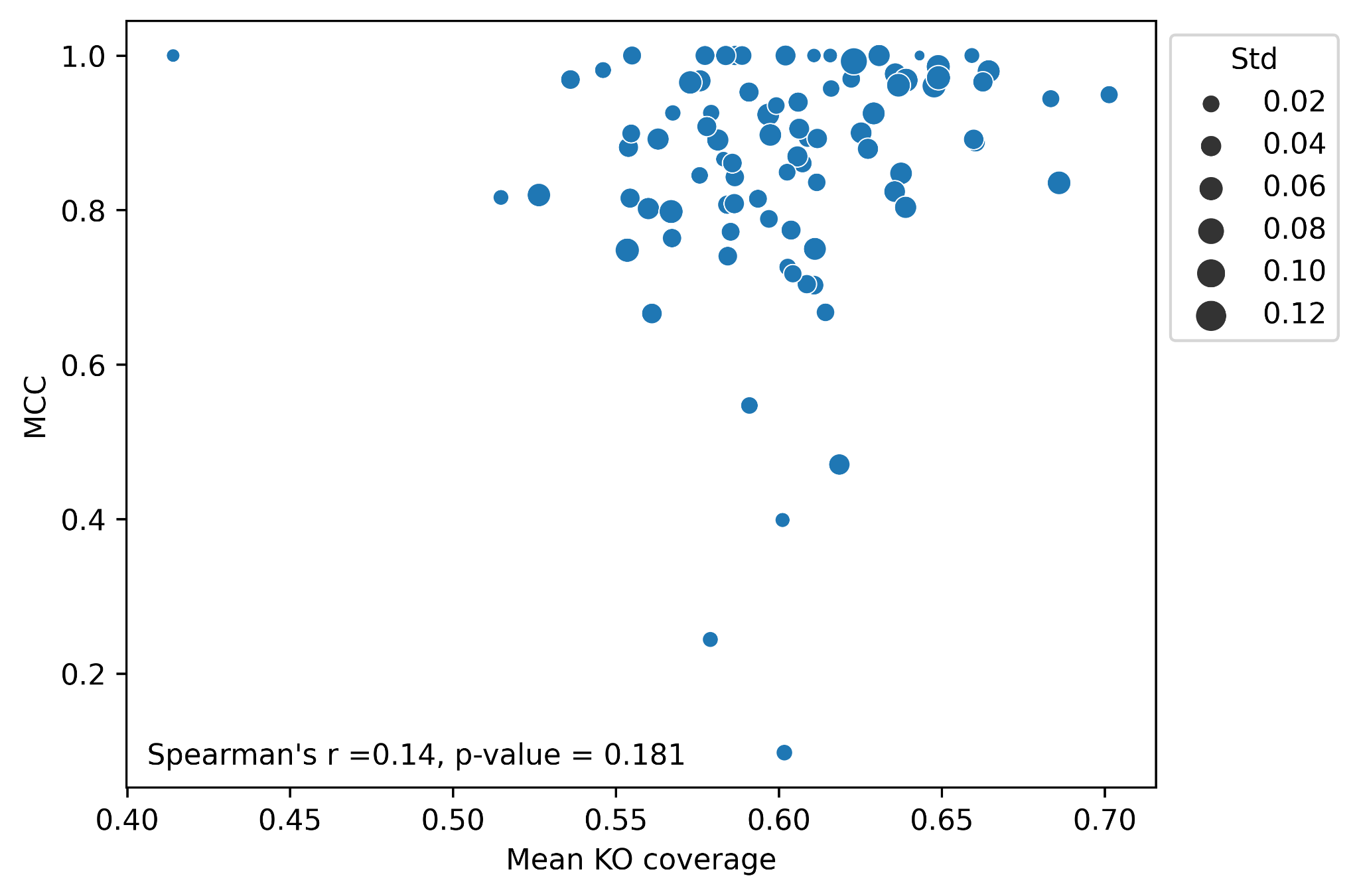


*Supplementary Figure S6.* Model performance (MCC) shows no correlation with Mean KO coverage of genomes per functional class. Dot size indicates KO coverage standard deviation.


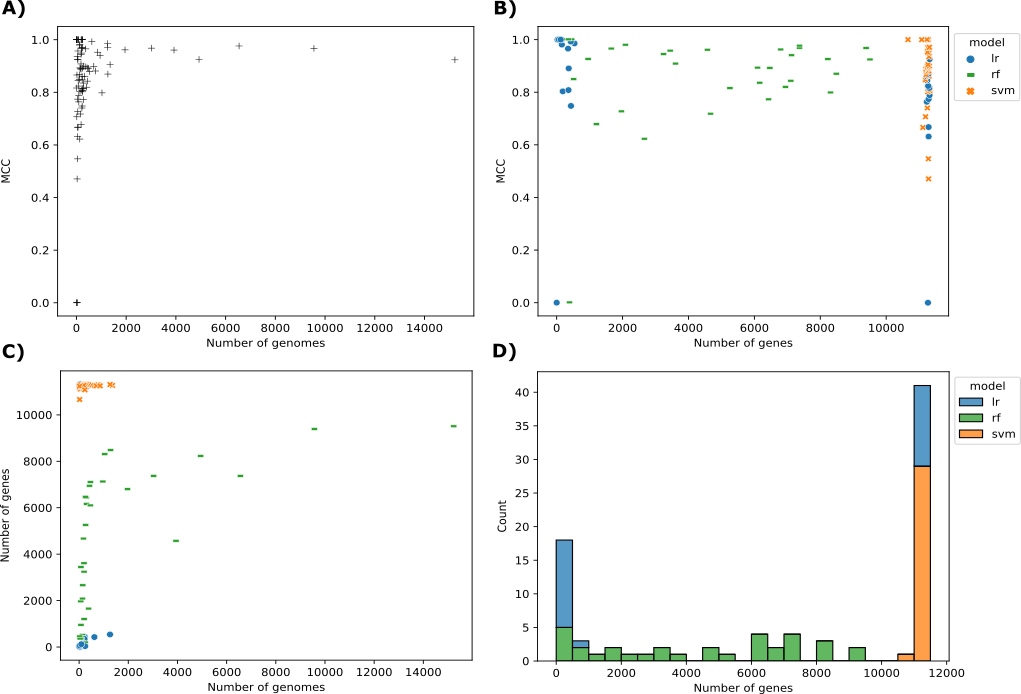


*Supplementary Figure S.7*. Distribution of classifiers’ MCC scores on the test set according to the number of genomes in the associated functional class (A) and the number of genes relevant for the classification (B). C) Number of relevant genes per genome number in each functional class. D) Number of genes considered relevant for the classification by the trained classifiers. The colour scale and marker types refer to the machine learning algorithms used for feature (gene) extraction.


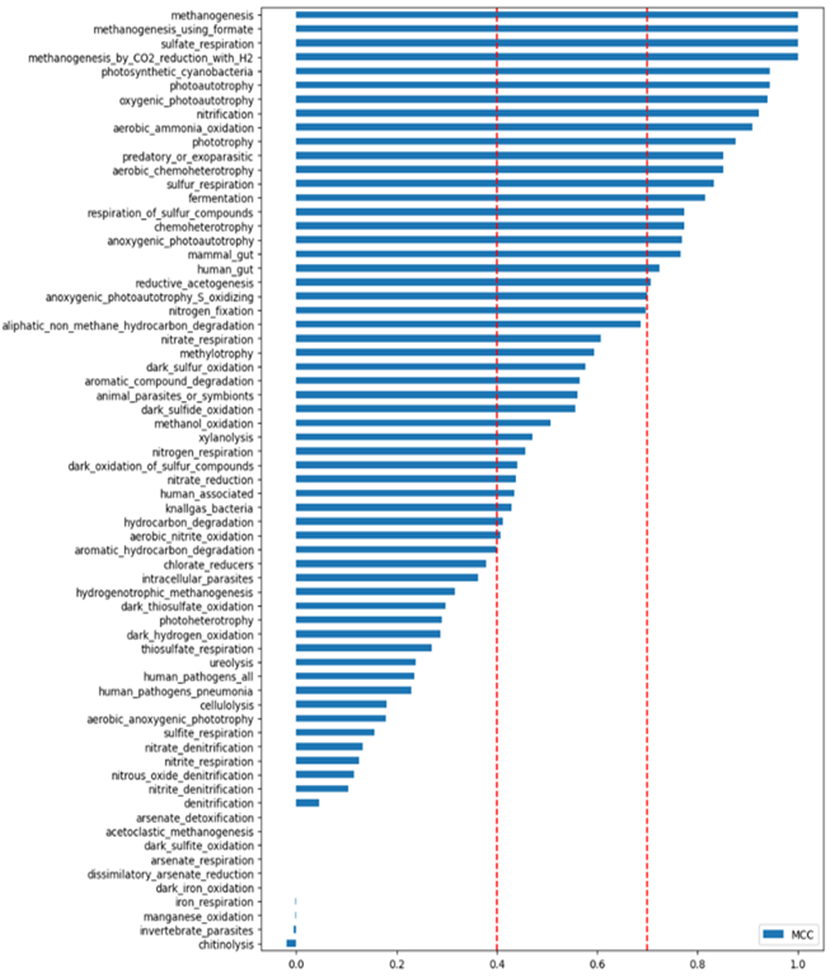


*Supplementary Figure S.8*. MCC scores of models on the independent dataset composed of 4,146 complete genomes for the 67 functional classes with true positives. Red lines represent the thresholds applied to evaluate the performance: MCC > 0.7 indicate good classifiers.

**A)**


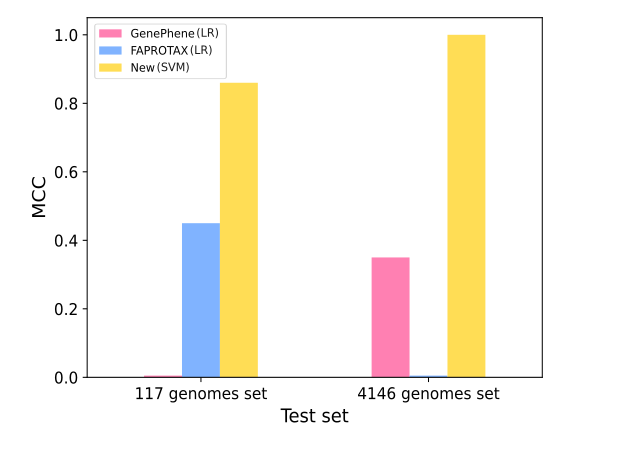


**B)**

| Training set version | | GenePhene | FAPROTAX-based | New |
| --- | --- | --- | --- | --- |
| Functional class size | | 7 | 8 | 15 |
| Chosen algorithm | | *l1*-Logistic regression | *l2*-Logistic regression | Support vector machines |
| Number of relevant genes | | 7 | 5492 | 4101 |
| MCC | 117 genomes set | 0 | 0.45 | 0.86 |
|  | 4146 genomes set | 0.35 | 0 | 1 |

*Supplementary Figure S.9*. A-B) Comparison of relevant information and performances of the three discussed acetoclastic methanogenesis classifiers on two independent datasets.
